# MTCH2 is a mitochondrial outer membrane protein insertase

**DOI:** 10.1126/science.add1856

**Published:** 2022-10-20

**Authors:** Alina Guna, Taylor A. Stevens, Alison J. Inglis, Joseph M. Replogle, Theodore K. Esantsi, Gayathri Muthukumar, Kelly C.L. Shaffer, Maxine L. Wang, Angela N. Pogson, Jeff J. Jones, Brett Lomenick, Tsui-Fen Chou, Jonathan S. Weissman, Rebecca M. Voorhees

**Affiliations:** 1 Whitehead Institute for Biomedical Research, Massachusetts Institute of Technology, Cambridge, MA 02142, USA; 2 Division of Biology and Biological Engineering, California Institute of Technology, 1200 E. California Ave., Pasadena, CA 91125, USA; 3 Medical Scientist Training Program, University of California, San Francisco, San Francisco, CA 94158, USA; 4 Tetrad Graduate Program, University of California, San Francisco, San Francisco, CA 94158, USA; 5 Howard Hughes Medical Institute, Massachusetts Institute of Technology, Cambridge, MA 02142, USA; 6 Department of Biology, Massachusetts Institute of Technology, Cambridge, MA 02142, USA; 7 Present address: Oncovalent Therapeutics 1290 Rancho Conejo Blvd Ste 101-F Thousand Oaks, CA 91320; 8 David H. Koch Institute for Integrative Cancer Research, Massachusetts Institute of Technology, Cambridge, MA,02142, USA

## Abstract

In the mitochondrial outer membrane, α-helical transmembrane proteins play critical roles in cytoplasmic-mitochondrial communication. Using genome-wide CRISPR screens, we identified MTCH2, and its paralog MTCH1, and showed that it is required for insertion of biophysically diverse tail-anchored (TA), signal-anchored, and multipass proteins, but not outer membrane β-barrel proteins. Purified MTCH2 was sufficient to mediate insertion into reconstituted proteoliposomes. Functional and mutational studies suggested that MTCH2 has evolved from a solute carrier transporter. MTCH2 uses membrane-embedded hydrophilic residues to function as a gatekeeper for the outer membrane, controlling mislocalization of TAs into the endoplasmic reticulum and modulating the sensitivity of leukemia cells to apoptosis. Our identification of MTCH2 as an insertase provided a mechanistic explanation for the diverse phenotypes and disease states associated with MTCH2 dysfunction.

## One-Sentence Summary

We showed that MTCH2 was both necessary and sufficient for insertion of diverse α-helical proteins into the mitochondrial outer membrane, and was the defining member of a family of insertases that have co-opted the SLC25 transporter fold.

Mitochondria are organelles of endosymbiotic origin that have evolved to play a central role in eukaryotic cell metabolism and signaling (1). Mitochondrial function and their ability to communicate with the cytosol depend on proteins embedded in the outer mitochondrial membrane. As a result, dysregulation of outer membrane protein function is associated with ageing and the pathogenesis of a variety of human diseases including Alzheimer’s, Parkinson’s, and many cancers (2–4). In mammals, the insertion of α-helical proteins into the outer membrane, a function that would not have been required in the ancestral endosymbiont, remains poorly understood (5). In yeast and trypanosomes the mitochondrial import protein 1 (Mim1) and pATOM36, respectively, have been implicated in this process (6, 7), but no clear homologs exist in mammalian mitochondria. One important class of α-helical outer membrane proteins are tail-anchored proteins (TAs), which are characterized by a single C-terminal transmembrane domain (TMD) and mediate diverse functions including apoptosis, innate immunity, and mitochondrial turnover and dynamics. Therefore, we set out to systematically identify and characterize the factors required for mitochondrial TA biogenesis in human cells.

**Fig. 1.**
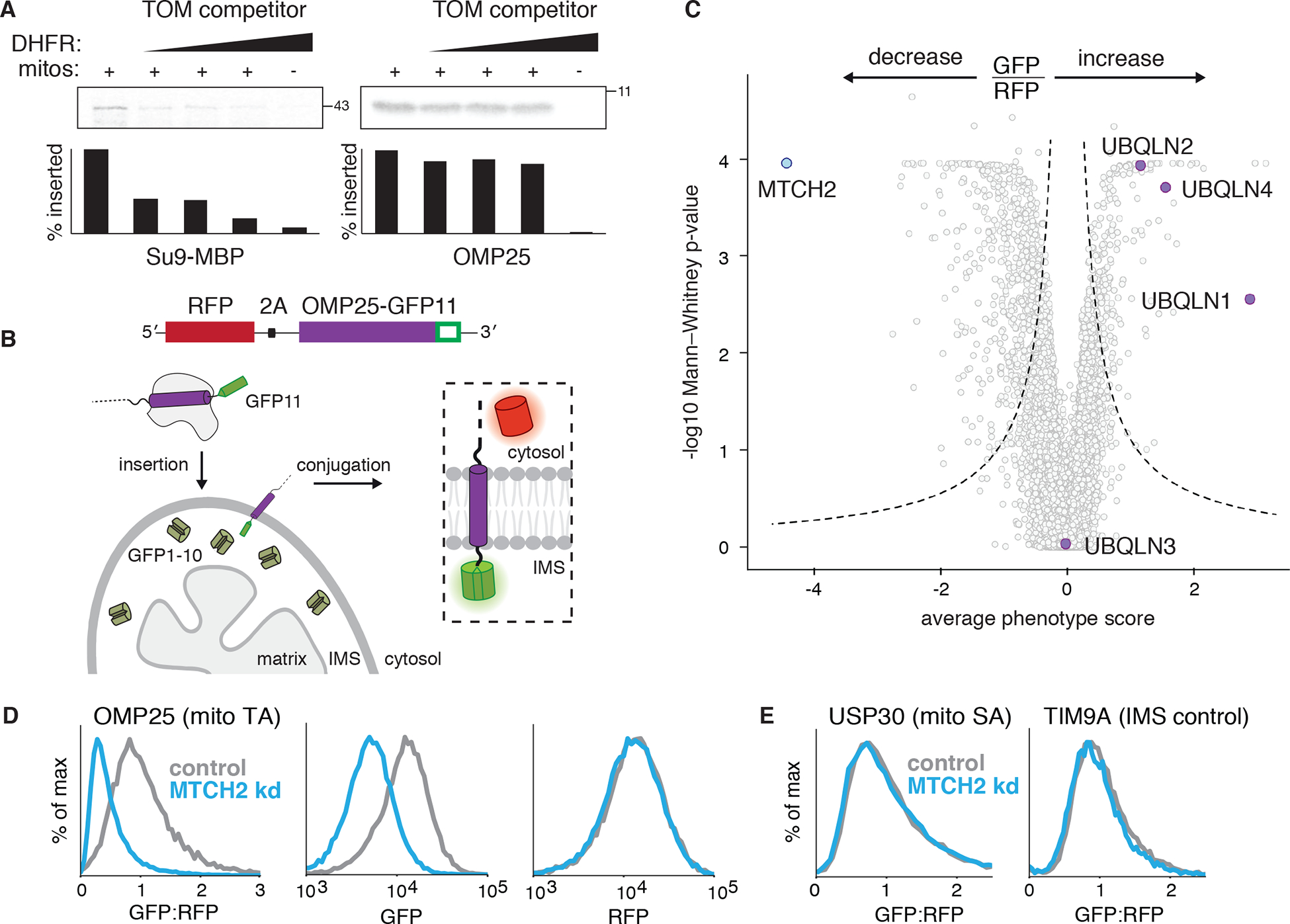
Systematic characterization of human mitochondrial TA biogenesis. **(A)** An ^35^S-methionine labelled TOM substrate (made from a fusion of the canonical TOM targeting sequence Su9 and the globular protein MBP) or OMP25 (a mitochondrial TA protein) were translated in rabbit reticulocyte lysate and released from the ribosome using puromycin. Competition assays were performed by incubation with purified mitochondria (see fig. S1) in the presence of increasing concentrations of a recombinant TOM competitor (Su9-DHFR). Mitochondrial insertion was assessed by protease protection and analyzed by SDS-PAGE and autoradiography. See also fig. S2. **(B)** Schematic of the split GFP reporter system used to specifically query integration of substrates into the outer mitochondrial membrane. A mitochondrial membrane protein fused to GFP11 is expressed in a cell constitutively expressing GFP1–10 in the intermembrane space (IMS) along with a translation normalization marker (RFP) Successful integration into the outer membrane results in complementation and GFP fluorescence. **(C)** Volcano plot of GFP:RFP stabilization phenotype for the three strongest sgRNAs versus Mann-Whitney p values from two independent replicates of a genome-wide CRISPRi screen using OMP25-GFP11. Individual genes are displayed in grey, and specific factors that increase or decrease OMP25 mitochondrial integration are highlighted and labelled. **(D)** Integration into mitochondria of the OMP25-GFP11 reporter described in (B) was assessed in K562 cells expressing a non-targeting (control) or MTCH2 knock down sgRNA. GFP fluorescence relative to the normalization marker RFP was determined by flow cytometry and displayed as a histogram. Individual channels are also shown. **(E)** Biogenesis of USP30-GFP11, an outer membrane resident signal anchored protein, and TIM9A-GFP11, an IMS localized protein, were assessed as in (D).

Using an in vitro competition assay we first showed that TA insertion does not strictly require the TOM complex, the major outer membrane translocase (Fig. 1A, fig. S1–2; (8)). Therefore, to enable CRISPR-based screens (9), we adapted and validated (Fig. 1B, fig. S3) a split-GFP reporter (10) to measure insertion of the model TA, OMP25, into mitochondria. Amongst hits that increased mitochondrial integration of OMP25 were the ER membrane protein complex (EMC) and the ubiquilin (UBQLN) chaperone family (Fig. 1C; fig. S4A). These results are consistent with the EMC serving as the major insertase for mislocalized mitochondrial TAs into the ER (fig. S4; (11)), and the UBQLNs’ role in degrading mislocalized mitochondrial TAs (12), leading to their accumulation in the cytosol (fig. S5).

Conversely, depletion of the outer membrane resident mitochondrial carrier homologue 2 (MTCH2) resulted in the most pronounced loss of OMP25 integration (Fig. 1D, fig. S6A). MTCH2 is a member of the solute carrier 25 (SLC25) family, integral membrane proteins best known for their role in transporting metabolites into the mitochondrial matrix, but its localization and sequence suggests its function has potentially diverged, and it has no known substrates or transporter activity (13). Further, loss of MTCH2 is associated with a variety of pleotropic phenotypes including defects in mitochondrial fusion, lipid homeostasis, and apoptosis (14–16). However, the underlying biochemical activity of MTCH2 is not known.

**Fig. 2.**
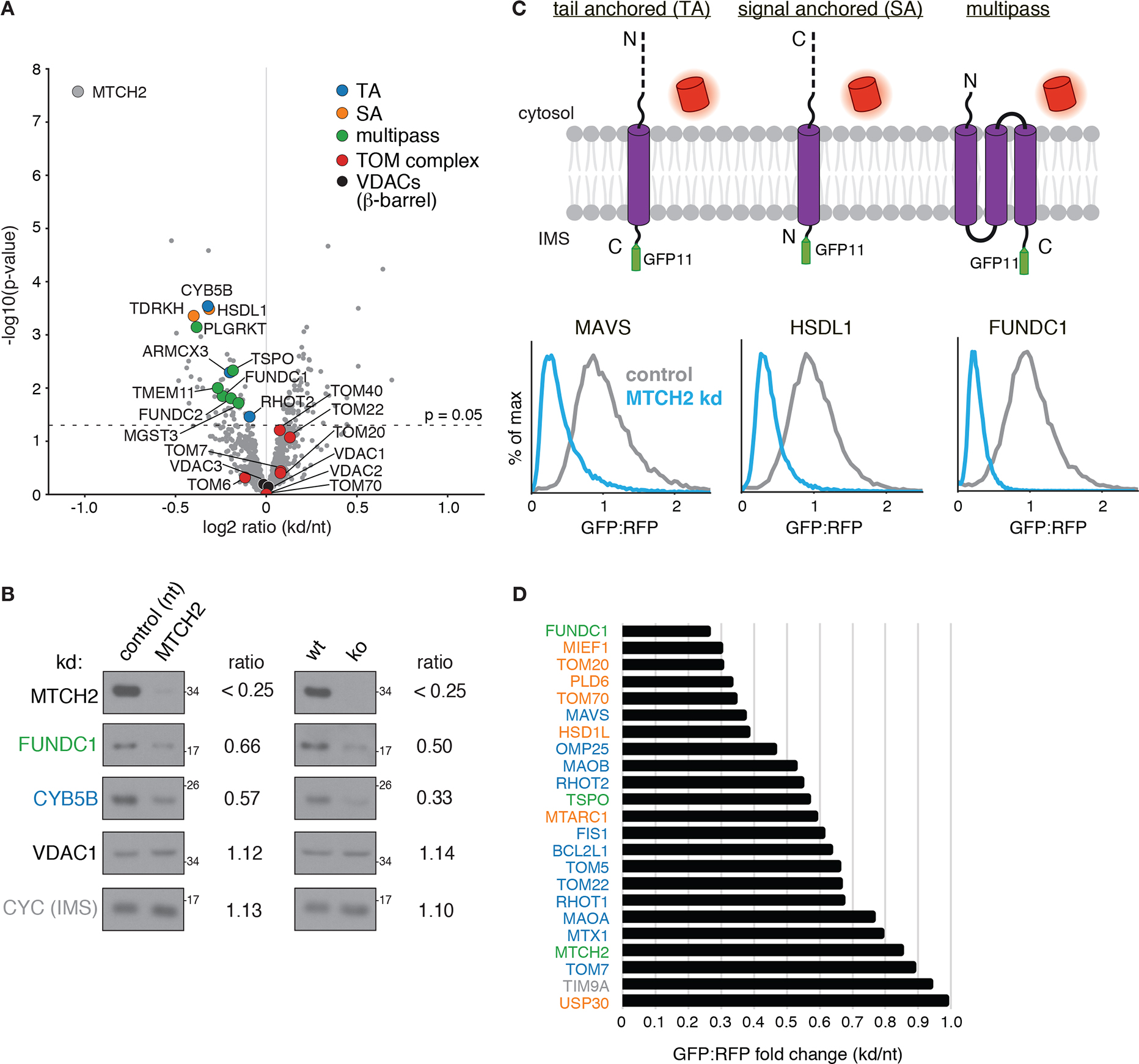
MTCH2 is required for mitochondrial outer membrane protein biogenesis. **(A)** Label-free mass spectrometry analysis of purified mitochondria isolated from K562 cells using a percoll gradient (fig. S1B) expressing a MTCH2 targeting sgRNA (kd) compared to a non-targeting control (nt). Displayed are proteins that across four biological replicates were statistically altered in MTCH2 depleted versus non-targeting guide expressing cells colored according to the indicated key (signal anchored: SA). **(B)** Immunoblotting of endogenous proteins in mitochondria isolated from MTCH2 depleted (kd) and control cells in (generated as in A; left), and wild type (wt) and MTCH2 knock out (ko) cells (right). Substrates are colored by topology based on the key shown in (A). Quantification of fold-change in depleted vs control cells is displayed as determined using a dilution series for each antibody. **(C)** Flow cytometry analysis of integration of outer membrane protein reporters using the split GFP system described in Fig. 1B. GFP fluorescence relative to an RFP expression control are displayed as histograms in MTCH2 knockdown versus non-targeting K562 CRISPRi cells. Displayed are representative examples of a TA, signal anchored (SA), and multipass membrane protein that have a MTCH2 dependent biogenesis defect. **(D)** Summary of dependence on MTCH2 for the indicated outer membrane substrates determined using the fluorescent reporter system shown in (C) and colored by topology based on the key in (A).

Because of the diverse phenotypes attributed to MTCH2, we excluded the possibility that dysregulation of lipogenesis (fig. S6B,C; (14)), the outer membrane, or general mitochondrial protein biogenesis (Fig. 1E) could explain the observed biogenesis defect on OMP25. We next sought to determine if MTCH2 could be playing a more general role in the biogenesis of other mitochondrial outer membrane proteins. Using a quantitative proteomics strategy, we compared the steady-state levels of endogenous proteins in mitochondria isolated from wildtype or MTCH2 depleted cells (Fig. 2A, fig. S7A, Tables S2–3). We identified several outer membrane α-helical TA, signal anchored, and multipass proteins, that were reproducibly decreased upon loss of MTCH2 (Fig. 2B). Because MTCH2 levels do not appreciably alter the mRNA levels for these proteins (fig. S7C; (17)), we concluded that the effects of MTCH2 on the mitochondrial outer membrane proteome must be occurring post-transcriptionally. To determine if MTCH2 exerts these effects specifically on biogenesis of nascent substrates, we tested a panel of mitochondrial proteins using our fluorescent reporter strategy (Fig. 1B). Consistent with the proteomics, MTCH2 affected the biogenesis of a functionally and biophysically diverse set of TA (18), signal anchored, and multipass proteins (Fig. 2C, D, fig. S8).

**Fig. 3.**
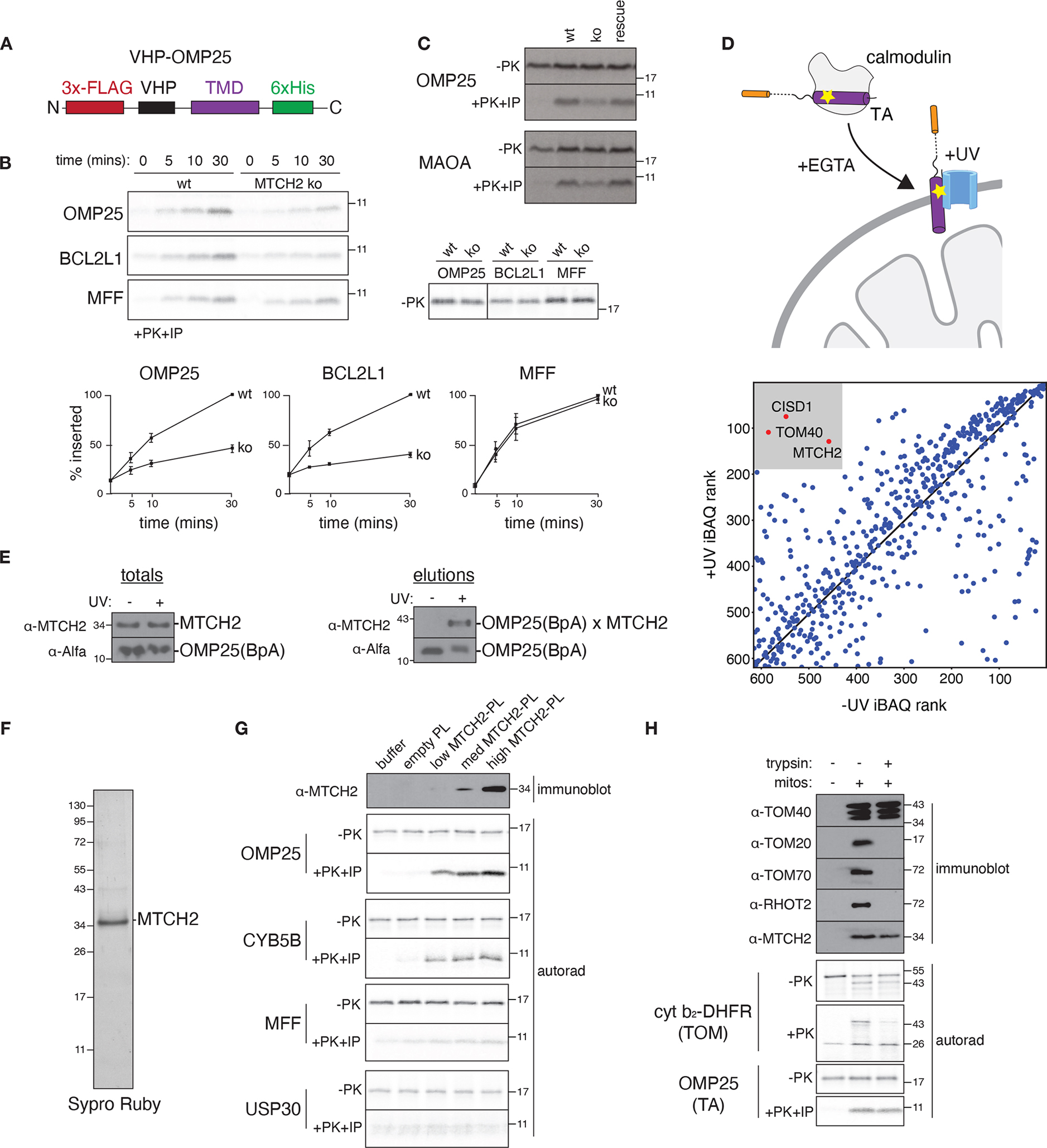
MTCH2 inserts diverse mitochondrial TAs into the outer membrane. **(A)** Schematic of the fusion between an inert N-terminal globular protein (VHP) and the TMDs of a panel of mitochondrial TAs (see also fig. S9) generated to probe TMD dependent insertion by MTCH2. **(B)** The indicated ^35^S-methionine labelled TA proteins were analyzed for in vitro insertion over time into mitochondria isolated from wild type (wt) or MTCH2 knockout (ko) K562 cells. Displayed are the samples prior to addition of protease (−PK; top right) and the protease protected fragment that has been affinity purified via a 6xHIS tag on the C-terminus of each substrate (+PK+IP; top left), ensuring insertion in the correct topology. (Bottom) Quantification of three biological replicates are plotted with error bars indicating one standard deviation at each time point. **(C)** As in (B) comparing insertion of the indicated TA proteins into wild type, MTCH2 ko, and MTCH2 ko + MTCH2 rescue mitochondria. **(D)** (Top) Schematic showing the photocrosslinking strategy. OMP25 containing the photoactivatable amino acid BpA within its TMD was expressed and purified from *E. coli* as a complex with calmodulin. OMP25^BpA^ was released from calmodulin by addition of EGTA in the presence of mitochondria purified from K562 cells using a percoll gradient (fig. S1B). Crosslinking was activated by UV-irradiation, and the resulting crosslinked species were affinity purified via the Alfa-tag on the N-terminus of OMP25^BpA^ for identification by mass spectrometry. (Bottom) All proteins identified by mass spectrometry were ranked by iBAQ abundance, and those specifically enriched in the UV compared to the -UV control are highlighted. Though TOM40 and CISD1 were identified, they were not significant hits in our screen (fig. S12), while TOM40 was not required for biogenesis both in vitro (Fig. 1A) and in cells (fig. S12B) **(E)** As in (D) with the resulting elution analyzed by immunoblotting to assess levels of crosslinked OMP25 ^BpA^-MTCH2. (**F)** MTCH2 was expressed and purified from human cells and analyzed by SDS-PAGE and Sypro-Ruby staining. **(G)** Following reconstitution (see fig. S13 for optimization of conditions), the recovered proteoliposomes were analyzed by immunoblotting for incorporation of MTCH2. Using a protease protection assay, the indicated MTCH2 dependent (OMP25, CYB5B) and independent (MFF, USP30) ^35^S methionine labelled substrates synthesized in rabbit reticulocyte lysate were tested for insertion into liposomes reconstituted with increasing amounts of purified MTCH2 compared to an empty control. The resulting protease protected fragments were immunoprecipitated, imaged by autoradiography (autorad). **(H)** Mitochondria from wt K562 cells were treated with trypsin and their ability to insert TOM (Su9-DHFR) or TA substrates (OMP25) was assayed by protease protection as in (A). The indicated outer membrane proteins were confirmed to be degraded in a trypsin-dependent manner by immunoblot, while MTCH2 remained largely intact.

Based on these experiments, we reasoned that MTCH2 may have evolved the ability to insert α-helical proteins into the outer membrane. To test this hypothesis, we focused on TA proteins, because they are the largest class of α-helical outer membrane proteins and adopt a uniform topology. Using an in vitro insertion assay with purified mitochondria (Fig. 3A, fig. S1C, fig. S9), we found that loss of MTCH2 affected the insertion of several mitochondrial TA proteins, but not unrelated intermembrane- or matrix-targeted controls (Fig. 3B, C and fig. S10, fig S11). Further, using site-specific crosslinking (Fig. 3D, Table S4; (19)), we demonstrated that MTCH2 physically associated with nascent substrates during their insertion (Fig. 3E, fig. S12).

Finally, to determine whether MTCH2 is sufficient for TA insertion, we purified MTCH2 (Fig. 3F) and optimized conditions for its reconstitution into liposomes (fig. S13). Using a panel of α-helical substrates, we show that purified MTCH2 specifically stimulated insertion of MTCH2 dependent, but not MTCH2 independent TAs and signal anchored proteins (Fig. 3G, fig. S14). To reconcile these results with earlier observations that trypsin-treated mitochondria remain competent for TA insertion, we found that in contrast to several subunits of the TOM complex, MTCH2 is largely trypsin resistant (Fig. 3H). Cumulatively, the requirement for MTCH2 in vivo and in vitro for TA insertion, together with its reconstituted insertase activity and physical association with substrates, rigorously establishes MTCH2 as an insertase for α-helical mitochondrial outer membrane proteins.

**Fig. 4.**
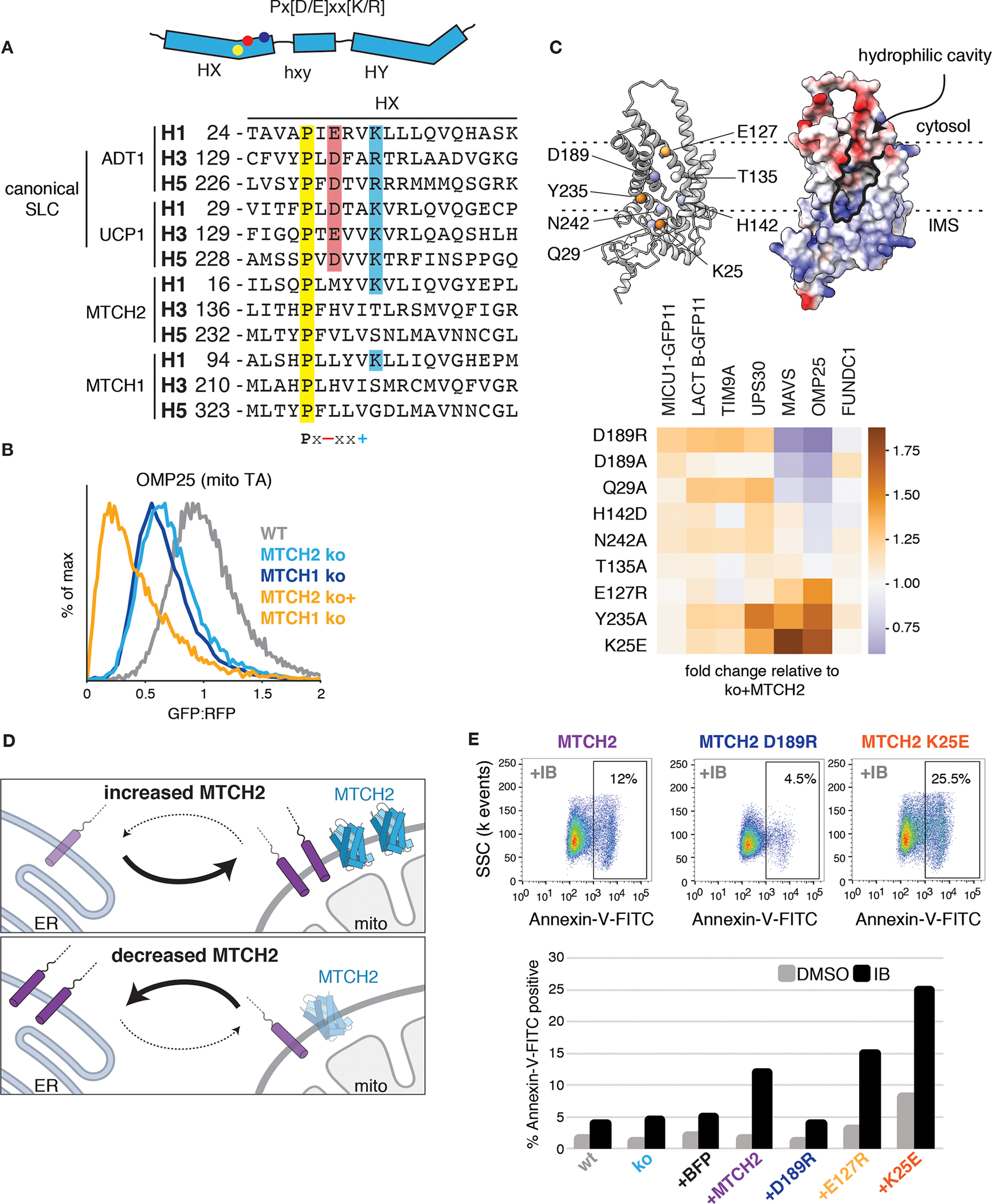
MTCH2 is a master regulator of outer membrane function. **(A)** (Top) SLC25 transporters are composed of three sets of two TMDs (six total). The location of the characteristic Px[D/E]xx[K/R] motif within a single SLC25 repeat is indicated. (Bottom) Sequence alignment of helices 1, 3, and 5 (with starting residues indicated) from two canonical inner membrane SLC25 transporters (ADT1, UCP1) and two diverged outer membrane SLC25 transporters (MTCH1, MTCH2), with residues from the Px[D/E]xx[K/R] motif highlighted. **(B)** Flow cytometry analysis of OMP25-GFP11 integration into the outer membrane using the reporter assay described in Fig. 1B. MTCH1 was depleted by transient knockout in either wild type (wt) or MTCH2 knock out (ko) cell lines. **(C)** (Top) AlphaFold2 predicted model of MTCH2 highlighting conserved polar and charged residues within the bilayer colored based on their effects on OMP25 shown below. (Bottom) using the reporter strategy shown in Fig. 1B, the indicated MTCH2 mutants, which alter the electrostatic potential of its TMDs, were tested for their effect on the indicated reporters (fig. S20). Depicted is a heat map summarizing the stimulation of each mutant relative to wild type MTCH2 on biogenesis of MTCH2 independent (MICU1, LACTB1, TIM9A, USP30) and dependent (MAVS, OMP25, FUNDC1) substrates. **(D)** Cell lines expressing GFP1–10 in the ER lumen were used to monitor mislocalization to the ER of mitochondrial TAs fused to a C-terminal GFP11. Table summarizing the analysis when either MTCH2 is depleted or overexpressed (data in fig. S20A, fig. S21, and fig. S22). **(E)** K562 cells expressing varying levels of MTCH2 or inactive (D189R) or hyperactive MTCH2 mutants (E127R or K25E; Fig. 4C) were treated with the chemotherapeutic imatinib mesylate (IB; 1 μM) or carrier (DMSO) for 72 hours. Apoptosis was assessed by staining with Annexin-V-FITC and analyzed by flow cytometry. Shown are representative dot plots displaying the fraction of apoptotic cells upon IB treatment expressing wt MTCH2 compared to in inactive (D189R) or hyperactive mutant (K25E) (Top) as well as a summary table for all MTCH2 constructs in IB vs carrier treated control.

Bioinformatic analysis reveals that in addition to MTCH2, other examples of SLC25 family members lacking canonical sequence motifs are found in both mitochondria and peroxisomes (Fig. 4A, fig. S15). Indeed, depletion of the close paralog MTCH1 (20), which is also localized to the mitochondrial outer membrane, had an additive effect to loss of MTCH2 on biogenesis of many mitochondrial TAs (Fig. 4B, fig. S17). This result is consistent with our genome-wide screen (fig. S17C) and the synthetic lethal relationship between MTCH1 and 2 (21). We therefore propose that MTCH1/2 are the founding members of a unique class of membrane protein insertases that exploit the SLC25 transporter fold (fig. S18).

In contrast to other solute carrier family members, in which the transmembrane helices close to form a pore that allows charged species to cross the membrane, the AlphaFold2 (22) predicted model of MTCH2 contains a prominent groove that is accessible to the membrane and lined with charged and polar residues (Fig. 4C, fig. S19). By introducing mutations at positions that altered the electrostatic potential of its intramembrane surfaces, we identified mutants that both diminish and enhance biogenesis of MTCH2-dependent but not independent substrates (Fig. 4C, fig. S19–20). We therefore concluded that MTCH2’s role in TA insertion relies on a hydrophilic surface within the bilayer.

Given MTCH2’s central role in mitochondrial TA biogenesis, we asked whether it may broadly affect cellular proteostasis. We find that indeed, depletion of MTCH2 leads to an increase in ER insertion of mitochondrial TAs, while MTCH2 overexpression leads to a commensurate decrease in their mistargeting to the ER (Fig. 4D, fig. S21–22). This effect was enhanced by further depleting ATP13A1 (19), an ER dislocase for mislocalized mitochondrial TAs (fig. S21C). These data suggest that MTCH2 is a central ‘gatekeeper’ for the mitochondrial outer membrane: MTCH2 levels and activity dictate the cytosolic reservoir of mitochondrial TAs, which then can be re-routed to the ER if unable to successful integrate into mitochondria (Fig. 4D).

Finally, considering that insertion of several MTCH2-dependent TAs play a central role in apoptosis, we reasoned that MTCH2 activity may affect cellular sensitivity to apoptotic stimuli. To test this, we overexpressed MTCH2 in human K562 cells, which are derived from a myelogenous leukemia cell line known to upregulate the anti-apoptotic TA, BCL2L1 (23). We treated cells with imatinib, a leukemia treatment which targets the BCR-ABL oncogene, and measured apoptosis. We found that while knockout of MTCH2 did not appreciably alter apoptosis propensity in this system, overexpression of wild type MTCH2 markedly sensitizes K562 cells to imatinib treatment (Fig. 4E). Critically, by expressing a series of MTCH2 mutants we found that this sensitization depends on MTCH2’s insertase activity.

In summary, we have demonstrated that MTCH2 is a defining member of a family of membrane protein insertases that are necessary and sufficient for insertion of TAs into human mitochondria. MTCH2’s insertase activity relies on a hydrophilic groove within the bilayer, an apparent example of convergent evolution of many membrane protein translocases including the EMC (24–26), Hrd1 (27), and YidC (28). A significant number of mitochondrial TAs are enriched in basic residues immediately C terminal to their TMDs (29), and may be particularly reliant on charged surfaces along their route into the membrane. MTCH2’s role also appears to extend to the integration of a broader class of α-helical proteins into the outer membrane, including signal anchored and multipass proteins. Homologs of MTCH2 are present in metazoan peroxisomes and its orthologs are found throughout holozoa, suggesting that the MTCH2 family has co-opted the SLC25 transporter fold to function in diverse biological membranes. The transition from a solute carrier, which mediates transport of small molecules across the membrane, to an insertase appears to have been enabled by the evolution of a membrane accessible hydrophilic groove absent from MTCH2’s SLC25 ancestors.

Previously, loss of MTCH2 has been reported to lead to a diverse range of phenotypes including dysregulation of mitophagy, mitochondrial fragmentation (14), recruitment of tBID (16), and altered lipid homeostasis (15), and was also identified in a recent genome-wide association study for Alzheimer’s disease (30–32). The identification of MTCH2 as a key gatekeeper for α-helical outer membrane proteins now provides a molecular explanation for its pleotropic phenotypes, many of which can be directly ascribed to defects in biogenesis of MTCH2 substrates.

## Supplementary Material

Guna et al supp.

Table S1

Table S2

Table S3

Table S4

## Data Availability

All data needed to evaluate the conclusions in this paper are present in the paper or the Supplementary Materials.
