# Supplementary material for: MTCH2 is a mitochondrial outer membrane protein insertase
Source: Science. Author manuscript; Available in PMC 2022 Nov 18. (PMC9674023; doi:10.1126/science.add1856)
Supplement: Guna et al supp. [file NIHMS1847457-supplement-Guna_et_al_supp_.pdf]

Supplementary Materials for

MTCH2 is a mitochondrial outer membrane protein insertase

Alina Guna*, Taylor A. Stevens*, Alison J. Inglis*, Joseph M. Replogle, Theodore K. Esantsi, Gayathri Muthukumar, Kelly C.L. Shaffer, Maxine L. Wang, Angela N. Pogson, Jeff J. Jones, Brett Lomenick, Tsui-Fen Chou, Jonathan S. Weissman†, and Rebecca M. Voorhees†

correspondence to: weissman@wi.mit.edu (J.S.W); voorhees@caltech.edu (R.M.V)

This PDF file includes:

Materials and Methods
Figs. S1 to S22
Captions for Tables S1 to S4
References

Other supplementary materials:

Tables S1 to S4 (.xlsx)

Materials and Methods

Plasmids and antibodies

Endogenous sequences used in this study for in vitro and in vivo analysis were sourced from UniProtKB/Swiss-Prot and included: squalene synthase isoform 1 (SQS/FDFT1; **Q6IAX1**), synaptojanin-2 binding protein (OMP25/SYNJBP; **P57105-1**), mitochondrial antiviral-signaling protein (MAVS; **Q7Z434-1**), mitochondrial import intermembrane translocase subunit Tim9 (TIM9; **Q9Y5J7-1**), vesicle associated membrane protein 2 (VAMP; **P51809-1**), FUN14 domain-containing protein 1 (FUNDC1; **Q8IVP5-1**), mitochondrial import receptor subunit TOM5 homolog (TOM5; **Q8N4H5-1**), mitochondrial import receptor subunit TOM22 homolog (TOM22; **Q9NS69-1**), ubiquitin carboxyl-terminal hydrolase 30 (USP30; **Q70CQ3-1**), apoptosis regulator BAX (BAX; **Q07812-1**), Bcl-2 homologous antagonist/killer (BAK1; **Q16611-1**), Bcl-2-like protein 1 (BCL2L1; **Q07817-1**), Cytochrome b5 type B (CYB5B; **O43169-1**), peptidyl-prolyl cis-trans isomerase FKBP8 (FKBP8; **Q14318-1),** mitochondrial fission factor (MFF; **Q9GZY8-1),** inactive hydroxysteroid dehydrogenase-like protein 1 (HSDL1; **Q3SXM5-1**), amine oxidase [flavin-containing] A (MAOA; **P21397-1**), amine oxidase [flavin-containing] B (MAOB; **P27338-1**), mitochondrial Rho GTPase 1 (RHOT1; **Q8IXI2-1**), mitochondrial Rho GTPase 2 (RHOT2; **Q8IXI1-1**), CDGSH iron-sulfur domain-containing protein 1 (CISD1; **Q9NZ45**), metaxin-1 (MTX1; **Q13505-3**), translocator protein (TSPO; **P30536)**, mitochondrial amidoxime-reducing component 1 (MTARC1; **Q5VT66-1**), mitochondrial cardiolipin hydrolase (PLD6; **Q8N2A8**), mitochondrial dynamics protein MID51 (MIEF1; **Q9NQG6-1**), mitochondrial import receptor subunit TOM20 homolog (TOM20; **Q15388**), mitochondrial import receptor subunit TOM7 homolog (TOM7; **Q9P0U1**), and mitochondrial carrier homolog 2 (MTCH2; **Q9Y6C9**).

Constructs for expression in rabbit reticulocyte lysate (RRL) used the SP64 vector as a backbone (Promega, Madison, WI). For in vitro insertion reactions, an N-terminal 3xFLAG tag and a C-terminal 6xHis tag were appended to the respective termini for affinity purification (see fig. S9A). Where noted, the transmembrane domains (TMDs) of TA proteins along with N- and C-terminal flanking sequences were instead conjugated to maltose binding protein (MBP) (*33*) or villin headpiece (VHP) domains as illustrated in fig. S9B. When monitoring insertion into the endoplasmic reticulum (fig. S4 and S21), constructs were modified to replace the 6xHis tag with a C-terminal opsin tag containing a glycosylation acceptor site which can be used as a proxy for insertion (*34*). TOM-dependent mitochondrial import substrates were designed by fusing dihydrofolate reductase (DHFR) to mitochondrial transit sequences from either *N. crassa* ATP synthase subunit 9 (residues 1–69) or *S. cerevisiae* cytochrome b_2 (residues 1–167), which direct import to the mitochondrial matrix or intermembrane space (IMS), respectively.

For expression in K562 cells, the basis for all constructs was a mammalian expression lenti-viral backbone containing a UCOE-EF-1α promoter and a 3′ WPRE element ((*35*); Addgene #135448). The exception was the dual fluorescent reporter used for the CRISPRi screen (RFP-P2A-OMP25-GFP11) which was integrated into an SFFV-tet3G backbone (*36*). The dual color reporter system used for in vivo experiments has been previously described (*37*, *38*). Note that the mCherry variant of RFP was used in all instances, but the simpler nomenclature of RFP is used in the text and figures. For complementation with the GFP1-10 system, the GFP11 tag

(RDHMVLHEYVNAAGIT) was appended to the appropriate terminus of the indicated protein as determined by predicted topology (see Fig. 2C). In order to express GFP1-10 in the ER lumen, the human calreticulin signal sequence was appended preceding GFP1-10-KDEL (*39*, *40*). For targeting to the intermembrane space of the mitochondria, either the targeting signal from MICU1 (residues 1–60) (*10*) or the targeting sequence from LACTB (residues 1–68) (*41*) was appended to the N terminus of GFP1-10. Expression of MTCH2 or indicated mutants was from a BFP-P2A-(MTCH2) cassette, allowing us to gate and sort for expressing cells.

The single sgRNA for MTCH2 (GACGGAGCCACCAAGCGACC) was generated by annealed oligo cloning of top and bottom oligonucleotides (Integrated DNA Technologies, Coralville, IA) into a lentiviral pU6-sgRNA EF-1α-Puro-T2A-BFP vector digested with BstXI/BlpI (Addgene, #84832). BFP was excised in certain sgRNA variants when the color interfered. Though most MTCH2 depletion experiments were done with a single guide that gave robust knockdown, key results were verified with an additional guide (GGGCTCACCGGGTCGCTTGG) to exclude possible off target effects. In many instances, we used a programmed dual sgRNA guide vector ((*42*); Addgene #140096) to allow for multiple genes to be depleted at once or to increase efficiency of knockdown. Dual guide pairs included MTCH2-ATP13A1 (GACGGAGCCACCAAGCGACC, GGGTAAAGCAGCCCGGCGAA), MTCH1-MTCH1 (GCGGCACCGCCGCGAGCCCA, GAGCCCAGGGCGCCACTTCC), and MTCH2-EMC2 (GACGGAGCCACCAAGCGACC, GGAGTACGCGTCCGGGCCAA). Transient knockout of MTCH1 was achieved by modifying a pLentiCRISPR backbone ((*43*), Addgene #102315) to express the following guides: GGACAACGCCCCGACCACTG and CTGCATCATCATCTCGTAGG. shRNA against TOM40 was purchased from Sigma Aldrich Mission shRNA with the targeting sequence CAAAGGGTTGAGTAACCATTT.

Constructs for recombinant bacterial protein expression were all cloned in the pQE plasmid (Qiagen, Valencia, CA). Su9-DHFR and CaM-3C-Alfa-Sec61β(2–60)-OMP25(112–145)F128Amber were cloned downstream of a His_{14}-*bd*SUMO tag. A GFP Nb fusion protein used in this manuscript was modified from a previously established construct ((*24*); Addgene #149336) to exclude the $SUMO^{Eu1}$ tag upstream of the GFP Nb. Constructs for the expression of $SENP^{EuB}$ ((*44*); Addgene #149333), *bd*SENP1 ((*45*); Gift from Dirk Görlich; Addgene #104962), and BirA ((*46*); Gift from Dirk Görlich, Addgene #149334), and for site-specific incorporation of BpA at the Amber codon position ((*47*); Addgene #31190) have all been previously described.

Constructs for generating human stable cell lines for recombinant protein expression used either the pHAGE2 plasmid (gift from Magnus A. Hoffmann and Pamela Bjorkman) for lentiviral integration into the Expi293F cell line or the pcDNA5/FRT/TO plasmid (Thermo Fisher Scientific, #V652020) for recombinase-mediated integration into Flp-In 293 T-REx cell line. MTCH2 or HA-MTCH2 were N-terminally fused with a GFP-$SUMO^{Eu1}$ tag and downstream of a doxycycline-inducible CMV promoter. The EMC3-GFP-P2A-RFP expression vector has been previously described (*24*).

All plasmids are available on request.

The following antibodies were used in this study: MTCH2 (ab113707, Abcam, UK); FUNDC1 (OAAB12808, Aviva systems biology, USA); CYB5B (HPA007893, Atlas antibodies, USA);

MIRO2 (RHOT2) (ab224089, Abcam, UK); CYC (4272, Cell signaling technology, USA); SYNJ2BP (OMP25) (15666-1-AP, Proteintech, USA); EMC3 (67205, Proteintech, USA); VDAC1 (sc-390996, Santa Cruz Biotech, USA); mitofilin (ab110329, Abcam, UK); SAMM50 (ab133709, Abcam, UK); ATP13A1 (16244-1-AP, Proteintech, USA); tubulin (T9026, Sigma-Aldrich, USA); UBQLN2 (WH0029978M3, Sigma-Aldrich, USA); TOM20 (sc-17764, Santa Cruz Biotech, USA); TOM40 (sc-365467, Santa Cruz Biotech, USA); TOM70 (14528-1-AP, Proteintech, USA). The ALFA tag was detected by coupling HRP to an ALFA nanobody (*48*). The Sec61β antibody was a gift from Ramanujan Hegde. Secondary antibodies used for immunoblotting were: goat anti-mouse- and anti-rabbit-HRP (#172-1011 and #170-6515, Bio-Rad, USA).

<u>Cell culture and cell line generation</u>

K562 cells were grown in RPMI-1640 with 25 mM HEPES, 2.0 g/L $NaHCO_3$, and 0.3 g/L L-glutamine supplemented with 10% FBS (or Tet System Approved FBS), 2 mM glutamine, 100 units/mL penicillin, and 100 µg/mL streptomycin. Cells were maintained at a confluency between 0.25×10^6–1×10^6 cells/mL. HEK293T cells were grown in DMEM supplemented with 10% FBS, 100 units/mL penicillin and 100 µg/mL streptomycin. All cell lines were grown at 37 °C.

For the expression of EMC3-GFP, Flp-In 293 T-Rex cells were purchased from Thermo Fisher Scientific (USA) (RRID: CVCL_U427) and grown in DMEM supplemented with 2 mM glutamine, 10% FBS, 15 µg/mL Blasticidine S, and 100 µg/mL Zeocin. The open reading frame to be integrated into the genomic FRT site was cloned into the pcDNA5/FRT/TO vector backbone and cell lines were generated according to the manufacturer's protocol. To allow for large scale growth, these cells were adapted to grow in suspension. Briefly, over the course of 10 days the FBS-supplemented DMEM was serially diluted with FreeStyle 293 Expression Medium (Thermo Fisher Scientific). Once growing in 100% FreeStyle Medium, the cells were transferred to 1–2 L roller bottles (Celltreat, USA) and grown in a shaking incubator operating at 8% CO_2 and rotating at 125 rpm. For the expression of MTCH2, lenti-viral infected inducible Expi293F suspension cells were grown in Expi293 expression media (Thermo Fisher Scientific) at 37 °C, 8% CO_2 and 125 rpm shaking in 1 L roller bottles with vented caps (Celltreat).

Three K562 cell lines were used as a basis for cell lines generated in this study: CRISPRi K562 cells expressing dCas9-BFP-KRAB (KOX1-derived) (*9*), K562-dCas9-BFP-KRAB Tet-On cells (*36*), and CRISPRi cells generated by stably expressing ZIM3 KRAB-dCas9-P2A-BFP from a UCOE-SFFV promoter (*17*). To generate cell lines with GFP1-10 in the mitochondrial IMS or ER lumen, virus was made from the respective constructs: LACTB(GFP1-10), MICU1(GFP1-10) or CalR(GFP1-10)-KDEL. CRISPRi K562 cells were infected with lenti-virus and sorted into 96-well plates as single cell clones using a Sony Cell Sorter (SH800S). After expansion, correct cell lines were confirmed by successful complementation with a construct targeted to the respective compartment, appended to a GFP11. To generate the cell line used for screening, lenti-virus containing MICU1(GFP1-10) and RFP-P2A-OMP25-GFP11 under a tet-inducible promoter were co-infected to one copy per cell in CRISPRi K562 Tet-On cells, single cell sorted, and verified by induction with doxycycline (100 ng/mL) and microscopy in conjunction with MitoTracker staining to confirm correct localization.

To generate MTCH2 knockout cell lines, K562 CRISPRi cells with or without MICU1(GFP1-10) were nucleofected with a MTCH2 targeting guide in the pX458 backbone (Addgene #48138) using the Lonza SF Cell Line 96-well Nucleofector Kit (V4SC-2096). The pX458 backbone was adapted to express two sgRNAs targeting MTCH2 [AGCCGACATGTCTCTAGTGG], [GGCTTTGCGAGTCTGAACGT]. Two days following nucleofection, GFP-positive cells were single cell sorted into 96-well plates. After colonies from single cells grew out, loss of MTCH2 was confirmed by immunoblotting. CRISPR-Cas9-induced genome edits were identified using the computational pipeline described in (*49*).

Lentivirus production

Lentivirus was generated by co-transfecting HEK293T cells with two packaging plasmids (pCMV-VSV-G and Δ8.9, Addgene #8454) and the desired transfer plasmid using TransIT-293 transfection reagent (Mirus). 48 hours after transfection, the supernatant was collected and flash frozen. In all instances, virus was rapidly thawed prior to transfection. Virus for the genome-wide CRISPRi screen was also generated using this method.

CRISPRi screen

The genome-scale CRISPRi screen was performed in duplicate as previously described (*9*, *50*). The hCRISPRi-v2 compact library (5 sgRNAs per gene, Addgene pooled library #83969) was transduced in duplicate into 330 million K562-CRISPRi-Tet-ON-((MICU1)-GFP1-10)-(tet-RFP-P2A-OMP25-GFP11) cells at multiplicity of infection (MOI) < 1 (percentage of transduced cells 48 hours after infection as measured by BFP positive cells: 30–35%). Cells were grown in 1 L of media in 1 L spinner flasks (Bellco, SKU: 1965-61010). 48 hours after spinfection with the genome-wide library, guide positive cells were selected with 1 μg/mL puromycin for three days. Following a 36 hour recovery, cells were induced with 100 ng/mL doxycycline for 36 hours and sorted on a FACS AriaII Fusion Cell Sorter. To ensure that the culture was maintained at an average coverage of more than 1000 cells per sgRNA, cells were diluted daily to 0.5×10^6 cells/mL.

During sorting, cells were gated for BFP (indicating a guide-positive cell), as well as GFP and RFP signal (successfully induced). Cells were sorted based on the GFP:RFP ratio of this final gated population. Roughly 40 million cells with either the highest (30%) or lowest (30%) RFP:GFP ratio were collected, pelleted and flash-frozen. Genomic DNA was purified using the Nucleospin Blood XL kit (Takara Bio, #740950.10) and amplified by index PCR with barcoded primers. The resulting guide library (~264 bp) was purified using SPRIbeads (SPRIselect Beckman Coulter #B23318). Sequencing was performed using an Illumina HiSeq2500 high throughput sequencer. Sequencing reads were aligned to the CRISPRi v2 library sequences, counted and quantified (*50*). Generation of negative control genes and calculation of phenotype scores and Mann–Whitney p-values was performed as described previously (*9*, *50*). Gene-level phenotypes and counts are available in Table S1.

Protein expression and purification

Su9-DHFR and CaM-3C-Alfa-Sec61β-OMP25(BpA)
The BL21(DE3) expression strain was used to express Su9-DHFR and CaM-3C-Alfa-Sec61β-OMP25(BpA) in LB media. Su9-DHFR expressing cultures were induced with 1 mM IPTG after an optical density of 0.6 was reached. After induction, Su9-DHFR was expressed at 37 °C for 3 hours. CaM-3C-Alfa-Sec61β-OMP25(BpA) was co-transformed with pEVOL-BpF and grown to an optical density of 0.2 followed by induction with 1% arabinose and the addition of 1 mM BpA (Bachem). Cells were then grown to an optical density of 0.6 followed by induction with 1 mM IPTG and expression at 25 °C for 6 hours. Cells were pelleted by centrifugation and resuspended in a lysis buffer containing 500 mM NaCl, 50 mM Tris pH 7.5, 10 mM imidazole, 5 mM β-ME.

For purification, *E. coli* resuspensions were supplemented with EDTA-free protease inhibitor tablets (Roche) and lysozyme prior to lysis by sonication. Lysate was clarified by centrifugation at 18,000 rpm for 30 minutes in an SS-34 rotor. Clarified lysate was incubated with NiNTA resin for 30 minutes while rolling at 4 °C. NiNTA resin was washed extensively with resuspension buffer, and then equilibrated with a SENP elution buffer containing 150 mM NaCl, 50 mM Tris pH 7.5, 10 mM imidazole, 5 mM β-ME, 10% glycerol. NiNTA resin was then incubated with *bd*SENP1 for 2 hours at 4 °C to release SUMO-cleaved protein from the resin.

For CaM-3C-Alfa-Sec61β-OMP25(BpA) the resuspension buffer included 1 mM $CaCl_2$, and SENP elution buffer contained 100 mM NaCl, 50 mM Tris pH 7.5, 10 mM imidazole, 5 mM β-ME, 1 mM $CaCl_2$. Additionally, protein was cleaved overnight with 3C protease following SENP1 elution. Cleaved protein was then concentrated to 250 µL and injected onto a Superdex 200 increase 10/300 GL size exclusion column equilibrated in a buffer containing 150 mM KoAc, 50 mM HEPES pH 7.4, 2 mM $MgAc_2$, 1 mM $CaCl_2$, 1 mM DTT, 10% glycerol. Protein-containing fractions were pooled, concentrated, aliquoted, and flash-frozen.

Biotinylated GFP-Nb and Alfa-NB
Expression and purification of all GFP and Alfa nanobody constructs as well as *bd*SENP1, BirA, and $SENP^{EuB}$ generally proceeded as follows: the NEB Express I^q expression strain was used with TB medium. Cultures were induced with 0.2 mM IPTG after an optical density of 2.0 was reached. Protein was expressed at 18 °C for 18–20 hours. Cells were pelleted by centrifugation and resuspended in a lysis buffer containing 50 mM Tris pH 7.5, 300 mM NaCl, 20 mM imidazole, 1 mM DTT, 1 mM PMSF. Cells were lysed by sonication and lysate was clarified by centrifugation at 18,000 rpm for 30 minutes in an SS-34 rotor. Clarified lysate was incubated with NiNTA resin for 1 hour while rolling at 4 °C. NiNTA resin was washed extensively with resuspension buffer and the eluted with a buffer containing 50 mM Tris pH 7.5, 300 mM NaCl, 500 mM imidazole, 250 mM sucrose. The imidazole was removed using a PD-10 desalting column (GE Healthcare, USA). The following protein-specific modifications were applied to the above protocol: the Rosetta-gami 2(DE3) expression strain was used to express His_{14}-Avi-SUMOstar-AlfaNb and expression time was limited to 6 hours at 18 °C. $SENP^{EuB}$ was limited to an expression time of 6 hours at 18 °C. *bd*SENP1 was expressed as a His_{14}-TEV- fusion and was cleaved with TEV protease overnight after buffer exchange and then run over NiNTA resin to remove the cleaved tag. The expression and purification of His_{14}-Avi-$SUMO^{Eu1}$-anti GFP nanobody, *bd*SENP1, BirA, and $SENP^{EuB}$ have all (*51*) been previously described (*24*, *44*).

All nanobody constructs used in this publication were biotinylated in a buffer containing 50 mM Tris pH 7.5, 100 mM NaCl, 12.5 mM $MgCl_2$, 10 mM ATP, 10 mM biotin and BirA at a 1:50 molar ratio to the nanobody substrate. Biotinylation reactions were incubated at 25 °C for 3 hours and then buffer exchanged in a PD-10 column equilibrated with a buffer containing 50 mM Tris pH 7.5, 200 mM NaCl, 1 mM DTT, 250 mM sucrose. Biotinylated protein was aliquoted and flash-frozen.

MTCH2 and EMC

We isolated MTCH2, GFP-$SUMO^{Eu1}$-HA-MTCH2, and EMC (via EMC3-GFP) under native conditions from detergent-solubilized cells using a biotinylated anti-GFP nanobody, expressed and purified as previously described (*24*). Cells were grown in 1 L roller bottles. For the MTCH2 lines, expression was induced for at least 48 hours with 10 µg/mL doxycycline. Cells were then harvested by centrifugation, washed with 1×PBS, and then pellets were weighed.

Purification generally proceeded as follows: cell pellets were resuspended at a ratio of 1 g to 10 mL hypotonic lysis buffer containing 10 mM HEPES pH 7.5, 10 mM KoAc, 0.15 mM $MgAc_2$, 0.5 mM DTT, supplemented with EDTA-free protease inhibitor tablets (Roche). The cell resuspension was incubated on ice for 10 minutes to allow cells to swell, and then lysed in a Dounce homogenizer with 10× strokes. The NaCl concentration was adjusted to 180 mM immediately after Dounce homogenization. Cell membranes were pelleted by centrifugation at 18,000 rcf in an SS-34 (28020TS, Thermo Fisher) rotor for 10 minutes. Supernatant was discarded and cell membranes were washed by resuspending and pelleting 2× in membrane wash buffer containing 10 mM HEPES/KOH pH 7.5, 200 mM NaCl, 0.15 mM $MgAc_2$, 0.5 mM DTT. The resulting pellet was resuspended at a ratio of 1 g (original cell pellet weight) to 6.8 mL solubilization buffer containing 50 mM HEPES pH 7.5, 200 mM NaCl, 2 mM $MgAc_2$, 1% deoxy-BigCHAP (DBC; Anatrace Cat # B310), 1 mM DTT, supplemented with EDTA-free protease inhibitor tablets (Roche). After 30 minutes of head-over-tail incubation with solubilization buffer, the lysate was cleared by centrifugation for 30 minutes at 4 °C and 18,000 rpm in a SS-34 rotor. The supernatant was then added to pre-equilibrated magnetic Streptavidin resin (Thermo Fisher) bound to biotinylated anti-GFP nanobody and blocked with free biotin. After 1 hour of binding while rolling at 4 °C, the resin was washed four times with wash buffer (solubilization buffer with 0.2% DBC). Resin was then incubated in wash buffer + 600 nM $SENP^{EuB}$ on ice for 2 hours to release SUMO-cleaved MTCH2 from the resin. Eluted samples were analyzed via SDS-PAGE with Sypro Ruby stain (Bio-Rad).

For EMC3-GFP, the whole cell pellet was solubilized in 1% DBC-containing buffer, without a hypotonic lysis and membrane washing step, and the GFP Nb was fused to a cleavable $SUMO^{Eu1}$ module. GFP-$SUMO^{Eu1}$-HA-MTCH2 was purified via a GFP Nb fused to a cleavable SUMOstar module.

Mitochondrial isolation, semi-permeabilized cells, and total membrane fractionation

Mitochondrial isolation for K562 cells was adapted from an established protocol (*52*). K562 cells were centrifuged at 220*g* for 5 minutes. Pellets were washed once in PBS and pelleted by spinning at 500*g* for 5 minutes. Pellets were resuspended in a homogenization buffer containing

210 mM mannitol, 70 mM sucrose, 5 mM HEPES pH 7.4, 10 mM EDTA, 1 mM PMSF, 2 mg/mL BSA. After incubating on ice for 10 minutes, cells were then lysed with a glass Dounce homogenizer with a tight-fitting pestle, or a Potter-Elvehjem homogenizer motor-driven at 1600 rpm for large scale purifications. Homogenized cells were pelleted at 1300*g* for 5 minutes to remove nuclei and unbroken cells, then the supernatant was transferred to a clean tube. This step was repeated twice. Nuclei-free homogenized cells were then centrifuged at 11,000*g* for 10 minutes. The supernatant was removed and the mitochondria-containing pellet was then resuspended in an isolation buffer containing 210 mM D-mannitol, 70 mM sucrose, 5 mM HEPES pH 7.4, 10 mM EDTA. Mitochondria were then pelleted and resuspended in fresh isolation buffer to wash away BSA and cytoplasmic proteins. After a final pelleting step, mitochondria were resuspended in a small volume (5–50 µL) of isolation buffer. To normalize mitochondrial samples, the protein concentration was measured using a Bradford assay.

For experiments using trypsin-treated mitochondria, the final two steps performed after pelleting mitochondria used import buffer containing 250 mM sucrose, 5 mM $MgAc_2$, 80 mM KOAc, and 20 mM HEPES. Mitochondria were then mixed with 0 or 50 µg/mL trypsin (Sigma-Aldrich #T1426) dissolved in import buffer and incubated on ice for 30 minutes. 50 µg/mL trypsin inhibitor (Sigma-Aldrich #T9128) and 1 mM PMSF were added to quench the reaction. Mitochondria were pelleted, washed once in import buffer + 5 µg/mL trypsin inhibitor, then resuspended in a concentrated volume prior to use in import experiments.

To further enrich mitochondrial samples for certain experiments, a percoll gradient was used. Isolation buffer density gradients were formed in three layers with 40%, 26%, and 12% percoll. Resuspended mitochondria were layered on top of the gradient. Gradients were centrifuged at 45,000*g* for 45 minutes in a TLS-55 rotor (Beckman Coulter). Pure mitochondria were retrieved from the 40%–26% percoll interface (see fig. S1B). Mitochondria were diluted 5-fold in isolation buffer, and then pelleted and washed in isolation buffer twice more.

HEK293T cells, either wild-type or an EMC5 knockout background were semi-permeabilized using standard methods (*12*). Briefly, 3×10^6 cells were collected and washed once with ice cold wash buffer containing 25 mM HEPES pH 7.4, 100 mM KOAc, 2 mM $MgAc_2$. The cells were resuspended in 1 mL SP buffer containing 25 mM Hepes pH 7.4, 100 mM KOAc, 2 mM $MgAc_2$, 50 µg/mL digitonin) and incubated on ice for 5 minutes. The semi-permeabilized cells were collected by centrifugation at 500*g* for 5 minutes at 4 °C, with the digitonin removed by washing three times. Finally, the cells were pelleted at 12,000*g* for 15 seconds and resuspended in 10 µL wash buffer. To test the integrity of the outer mitochondria membrane as in fig. S1A, SP cells and purified mitochondria (as described above) were incubated with the indicated amount of proteinase K (PK). Following quenching, the resulting reaction was subjected to blotting against mitofilin, an intermembrane localized protein which should not be accessible by PK when the outer mitochondrial membrane is intact.

To fractionate total cellular membranes from cytosol, 4 million K562 cells were pelleted by spinning at 500*g* for 5 minutes, then washed 1× in PBS and pelleted again. The washed pellet was then resuspended in 150 µL of a digitonin lysis buffer containing 50 mM HEPES pH 7.5, 10 mM KAc, 2 mM $Mg(Ac)_2$, 1 mM DTT, 0.05% digitonin, and supplemented with EDTA-free protease inhibitor tablets (Roche). The resuspension was incubated on ice for 10 minutes to

allow cells to swell, and then passed through a 27 G ¼" needle attached to a syringe 12×. The sample was then adjusted to 0.19 M NaCl, and centrifuged for 15 minutes at 18000*g* in a cold benchtop centrifuge. The cytoplasm-containing supernatant was removed and multiple samples were normalized via UV absorbance at 280 nm. The pellet was washed in digitonin lysis buffer + 0.19 NaCl. The wash pellet was resuspended in a solubilization buffer containing 50 mM HEPES pH 7.5, 200 mM NaCl, 2 mM $Mg(Ac)_2$, 1 mM DTT, 1% GDN, and supplemented with EDTA-free protease inhibitor tablets (Roche). After incubation on ice for 30 minutes, the sample was centrifuged for 10 minutes at 18000g in a cold benchtop centrifuge. The solubilized membrane containing supernatant was removed and multiple samples were normalized via UV absorbance at 280 nm.

<u>In vitro translation and insertion</u>

In vitro translations were carried out in rabbit reticulocyte lysate (RRL) as previously described (*38*). Constructs for in vitro translation reactions were based on the SP64 vector (Promega, USA). Templates for transcription were generated by PCR, with primers binding and upstream of the SP64 promoter and roughly 200 bp downstream of the stop codon (*53*). Following transcription at 37 °C for 1.5 hours, reactions were used directly in a translation reaction. Substrates were translated for 15–30 minutes at 32 °C in the presence of radioactive ^{35}S-methionine. Prior to addition of mitochondria or semi-permeabilized cells, 1 mM puromycin was added to prevent further synthesis.

Mitochondrial insertion reactions used isolated mitochondria, prepared as described above. Insertion reactions were performed by diluting 4 µL of a puromycin treated translation reaction in 50 µL of import buffer (250 mM sucrose, 5 mM $Mg(Ac)_2$, 80 mM KAc, 20 mM HEPES pH 7.4, 2.5 mM ATP, 15 mM succinate) with 15 µg of purified mitochondria and further incubating at 32 °C for 30 minutes. For competition experiments in Fig. 1A and fig. S2, insertion reactions were carried out in the presence of 1, 2, or 5 µM recombinant Su9-DHFR, and 5 µM methotrexate (BP266510, Fisher Chemicals, USA).

Protease digestions were initiated by the addition of proteinase K at 0.25 mg/mL, and reactions were then incubated on ice for 1 hour. Reactions were quenched by the addition of 5 mM PMSF in DMSO, followed by transfer to boiling 1% SDS (final concentration) in 0.1 M Tris/HCl pH 8.0. His-tagged protected fragments were enriched by incubating with NiNTA resin in IP buffer (50 mM HEPES pH 7.5, 500 mM NaCl, 10 mM imidazole, 1% Triton). Proteinase K-digested reactions were diluted to 1 mL and mixed with 10 µL resin, then incubated with end-over-end mixing for 1.5 hours at 4 °C. The resin was further washed with 3 × 1 mL IP buffer, and the products eluted from the resin in sample buffer containing 50 mM EDTA pH 8.0.

Insertion reactions with semi-permeabilized (SP) cells used a ratio of 1 µL cells per 10 µL translation reaction. To verify insertion of substrates into the ER as indicated by glycosylation, a tripeptide competitor of glycosylation (Asn-Tyr-Thr) was added at 50 µM when indicated.

Mass spectrometry

For the crosslinking-IP samples, TCA-precipitated pellets were resuspended in a buffer containing 8 M Urea and 100 mM Tris pH 8.5. The sample was reduced by incubation with 3 mM TCEP for 20 minutes, then alkylated by incubation with 10 mM iodoacetamide for 15 minutes, all at room temperature. The sample was then digested with 2 ng/µL LysC for 4 hours at room temperature, diluted 4-fold with 100 mM Tris pH 8.5, and $CaCl_2$ was added to 1 mM. The sample was then digested with 4 ng/µL trypsin overnight at room temperature. Samples were acidified by adding trifluoroacetic acid to 0.5%, desalted using Pierce C18 spin columns (Pierce), lyophilized, and then resuspended in 2% acetonitrile, 0.2% formic acid.

For the mitochondrial proteomics experiments, the S-trap sample preparation kit (ProtiFi) was used according to the manufacturer's instructions. Sample was digested on the S-trap column with 1 µg Trypsin per 10 µg protein overnight at 37°C. In addition to the provided S-trap sample preparation protocol, a final elution step with 70% acetonitrile, 1% formic acid was added. Eluted peptides were lyophilized, and then resuspended in 2% acetonitrile, 0.2% formic acid. The peptide concentration was determined with a Quantitative Fluorometric Peptide Assay (Pierce) kit.

LC-MS/MS analysis for the crosslinking-IP experiment (Fig. 3D, Table S4) was performed with an EASY-nLC 1200 (Thermo Fisher Scientific, San Jose, CA) coupled to a Q Exactive HF hybrid quadrupole-Orbitrap mass spectrometer (Thermo Fisher Scientific). Peptides were separated on an Aurora UHPLC Column (25 cm × 75 µm, 1.6 µm C18, AUR2-25075C18A, Ion Opticks) with a flow rate of 0.35 µL/min for a total duration of 75 min and ionized at 1.8 kV in the positive ion mode. The gradient was composed of 2%–6% solvent B (3.5 min), 6%–25% B (42 min), 25%–40% B (14.5 min), and 40%–98% B (15 min); solvent A: 2% ACN and 0.2% formic acid; solvent B: 80% ACN and 0.2% formic acid. MS1 scans were acquired at the resolution of 60,000 from 375 to 1500 m/z, AGC target 3e6, and maximum injection time 15 ms. The 12 most abundant ions were targeted for MS2 scans acquired at a resolution of 30,000, AGC target 1e5, maximum injection time of 60 ms, and normalized collision energy of 28. Dynamic exclusion was set to 30 s and ions with charge +1, +7, +8 and >+8 were excluded. The temperature of ion transfer tube was 275 °C and the S-lens RF level was set to 60. MS2 fragmentation spectra were searched with SEQUEST running within Proteome Discoverer (version 2.5, Thermo Fisher Scientific) against the UniProt human reference proteome comprised of 79,052 proteins covering 20,577 genes (UP000005640). The maximum missed cleavages was set to 2. Dynamic modifications were set to oxidation on methionine (M, +15.995 Da), deamidation on asparagine and glutamine (N and Q, +0.984 Da), phosphorylation of serine and threonine (S and T, +79.966 Da), protein N-terminal acetylation (+42.011 Da), and protein N-terminal Met-loss (-131.040 Da). Carbamidomethylation on cysteine residues (C, +57.021 Da) was set as a fixed modification. The maximum parental mass error was set to 10 ppm, and the MS2 mass tolerance was set to 0.03 Da. The false discovery threshold was set strictly to 0.01 using the Percolator Node validated by q-value. The relative abundance of parental peptides was calculated by integration of the area under the curve of the MS1 peaks using the Minora LFQ node.

To identify enriched proteins, proteins that were detected in both samples were ranked by iBAQ intensity within each sample, and enrichment was assessed based on the difference between the +UV and the -UV iBAQ rank. The final results of this analysis are listed in Table S4.

LC-MS/MS analysis to assess differences in protein content between percoll gradient-enriched mitochondria derived from K562 wild-type or MTCH2-depleted cells (Fig. 2A, Table S2) was performed with an EASY-nLC 1200 (Thermo Fisher Scientific) coupled to an Orbitrap Eclipse Tribrid mass spectrometer (Thermo Fisher Scientific). Peptides were separated on an Aurora UHPLC Column (25 cm × 75 µm, 1.6 µm C18, AUR2-25075C18A, Ion Opticks) with a flow rate of 0.35 µL/min for a total duration of 75 min and ionized at 1.8 kV in the positive ion mode. The gradient was composed of 2%–6% solvent B (3.5 min), 6%–25% B (42 min), 25%–40% B (14.5 min), and 40%–98% B (15 min); solvent A: 2% ACN and 0.2% formic acid; solvent B: 80% ACN and 0.2% formic acid. MS1 scans were acquired at the resolution of 120,000 from 350 to 1,600 m/z, AGC target 1e6, and maximum injection time of 50 ms. MS2 scans were acquired in the ion trap using fast scan rate on precursors with 2–7 charge states and quadrupole isolation mode (isolation window: 0.7 m/z) with higher-energy collisional dissociation (HCD, 30%) activation type. Dynamic exclusion was set to 30 s. The temperature of ion transfer tube was 300 °C and the S-lens RF level was set to 30. MS2 fragmentation spectra were searched with SEQUEST running within Proteome Discoverer (version 2.5, Thermo Fisher Scientific) against the reviewed sequences from the UniProt human reference proteome comprised of 20,383 proteins (UP000005640). The maximum missed cleavages were set to 2. Dynamic modifications were set to oxidation on methionine (M, +15.995 Da), deamidation (N and Q, +0.984 Da), protein N-terminal acetylation (+42.011 Da) and protein N-terminal Met-loss (-131.040 Da). Carbamidomethylation on cysteine residues (C, +57.021 Da) was set as a fixed modification. The maximum parental mass error was set to 10 ppm, and the MS2 mass tolerance was set to 0.6 Da. The false discovery threshold was set strictly to 0.01 using the Percolator Node validated by q-value. The relative abundance of parental peptides was calculated by integration of the area under the curve of the MS1 peaks using the Minora LFQ node.

LFQ was then performed with the Minora feature detector, feature mapper, and precursor ions quantifier nodes. Retention time alignment was performed with maximum RT shift of 5 min and a minimum S/N threshold of 10. Quantified peptides included unique + razor, protein groups were considered for peptide uniqueness, shared Quan results were not used, Quan results with missing values were not rejected, and precursor abundance was based on extracted ion intensity. Imputation was performed using a low abundance resampling method at the peptide level. The quantitative proteomics data was exported from ProteomeDiscoverer as an excel file and the MitoCoP database (*54*) was used to remove all non-mitochondrial proteins from the dataset prior to statistical analysis using the R statistical computing environment (R version 4.0.2). Protein abundances were normalized between samples with a random forest (R randomForest version 4.6-14) regression (*55*), then modeled for expression differences (R limma version 3.44.3) using the linear model fit, with fold changes calculated as a log2 difference. Proteins and peptides identified are listed in Table S2.

LC-MS/MS analysis to assess differences in protein content between crude isolated mitochondria derived from K562 wild-type or MTCH2-depleted cells (fig. S7A, Table S3) was performed similarly to the percoll-enriched mitochondrial samples with the following changes: peptides

were separated on an Aurora UHPLC Column (25 cm × 75 µm, 1.7 µm C18, AUR3-25075C18, IonOpticks) with a flow rate of 0.35 µL/min for a total duration of 75 min and ionized at 1.6 kV in the positive ion mode. MS2 scans were acquired in the ion trap using fast scan rate on precursors with 2–7 charge states and quadrupole isolation mode (isolation window: 1.2 m/z) with higher-energy collisional dissociation (HCD, 30%) activation type. Dynamic exclusion was set to 15 s. For LFQ, Retention time alignment was performed with maximum RT shift of 2 min and a minimum S/N threshold of 5.

Volcano plot figures were generated in Python using the matplotlib package. Figures that specify submitochondrial localization are based on annotations from Mitocarta 3.0 (*56*), with the following modifications: subcellular localizations for TDRKH, HSDL1, and ARMC1 were added manually based on published data (*57*, *58*), TMEM11 is annotated as IM in Mitocarta 3.0; however, a recent preprint provides evidence that TMEM11 is largely in the OM, so this gene was included in analysis of OM proteins in our proteomic data (*59*).

Photo-crosslinking of recombinant substrate with isolated mitochondria

An insertion reaction was prepared in a buffer containing 100 mM KAc, 50 mM HEPES pH 7.4, 2 mM $Mg(Ac)_2$, 0.5 mM $CaCl_2$. Percoll-gradient enriched mitochondria were added to a final protein concentration of 0.2 mg/mL and CaM-3C-Alfa-Sec61β-OMP25(BpA) was added to a final concentration of 1 µg/mL. Insertion was initiated by the addition of 2 mM EGTA. The insertion reaction was split into -UV and +UV samples. The +UV sample was transferred to a prechilled 6-well plate and left on top of an ice-cooled aluminum block ~8 cm under a UVP B-100 lamp for 10 minutes.

Both samples were mixed with 10× volumes of IP buffer containing 100 mM KAc, 50 mM HEPES pH 7.4, 2 mM $Mg(Ac)_2$, and 1% Triton X-100. After incubating on ice for 10 minutes, samples were centrifuged at 18,000 rpm. Supernatant was mixed with 10 µL packed streptavidin agarose resin (Thermo Fisher Scientific Cat. #20357) which had been functionalized with biotinylated Alfa-Nb, blocked with free biotin, and equilibrated in IP buffer. After 1 hour of binding head-over-tail at 4 °C, the unbound fraction was removed, and the resin was then washed 2× with 1 mL IP wash buffer, 2× with 1 mL IP wash buffer + 0.5 M NaCl, and 2× with 1 mL IP wash buffer. Resin was then incubated with 10 µL IP wash buffer + 300 nM SUMOstar protease (LifeSensors Cat # SP4110) for 30 minutes on ice. 1 mL IP wash buffer was added to protease treated resin and incubated for 5 minutes on ice. Eluted protein was then removed from resin and TCA precipitated by adding 1:10 volume 100% TCA and incubating on ice for 10 minutes, followed by centrifuging at max speed in a chilled benchtop centrifuge for 10 minutes. The pellet was then washed 2× with ice-cold acetone and prepared for mass spectrometry as described below.

Samples used for immunoblotting were prepared with the following differences: isolated mitochondria were not purified on a percoll gradient; Pierce magnetic streptavidin resin was used (Thermo Fisher Scientific Cat. #88817), an IP buffer containing 200 mM NaCl, 50 mM HEPES pH 7.4, 1% Triton X-100 was used for solubilization; 4 × 1 mL wash steps; and an elution buffer containing 200 mM NaCl, 50 mM HEPES pH 7.4, and 0.05% Triton X-100 was used for 2×

wash steps and then elution was performed in the same buffer + 300 nM SUMOstar in a 10 μL volume.

Proteoliposome reconstitutions and insertions

Reconstitutions of protein into liposomes were similar to previously described methods (*38*, *60*). The following phospholipids were obtained from Avanti Polar Lipids: phosphatidyl-choline (PC) and phosphatidyl-ethanolamine (PE) from egg, and synthetic 1,2-dioleoyl-sn-glycero-3-phosphoethanolamine-N-lissamine rhodamine B (Rh-PE). The liposome mixture contained PC:PE:Rh-PE at a mass ratio of 8:1.9:0.1. Rh-PE was used to monitor recovery throughout the reconstitutions and for quantification. Lipids were mixed at the indicated ratio as chloroform stocks, adjusted to 10 mM DTT and dried by centrifugation under vacuum overnight. The resulting lipid film was rehydrated to a final concentration of 20 mg/mL in lipid buffer (15% glycerol, 50 mM HEPES pH 7.4) and mixed for 8 hours at 25 °C until a homogenous mixture was achieved. Lipids were then diluted with more lipid buffer and supplemented with DBC to produce a lipid/DBC mixture containing 2% DBC and 10 mg/mL lipids. BioBeads-SM2 (Bio-Rad) were prepared by activation with methanol, washing thoroughly with distilled water, and then were resuspended in water into a final slurry where they occupied 50% of the final volume. For reconstitutions, excess liquid was removed from BioBeads by aspiration just before use. Reconstitutions using purified EMC and MTCH2 in 0.25% DBC were obtained as described below. In initial experiments, we determined the relative concentration of purified MTCH2 compared with the amount in isolated mitochondria from K562 cells. Different dilutions of purified MTCH2 were mixed with constant amounts of lipids and adjusted to a final buffer concentration of 100 mM NaCl, 25 mM HEPES pH 7.4, 2 mM $MgCl_2$, 0.8% DBC. Liposomes were made using the same buffer and detergent conditions. A standard 100 μL reaction contained 10-40 μL purified MTCH2, 20 μL of the 10 mg/mL lipid/DBC mixture, and the remaining volume made up with buffer, salts, and detergent. The molar ratio of lipid:protein used in reconstitutions was 5500:1, 2500:1 and 1500:1 depending for the high, medium, or low MTCH2-PL conditions, respectively. This protein/lipid/detergent mixture was added to 120–140 μL BioBeads in 1.5 mL Eppendorf tubes. The slurry was mixed in a thermomixer for 18 hours at 4 °C. In some cases, the fluid phase was removed and added to another 1.5 mL Eppendorf tube containing 120 μL Biobeads, and mixed in a thermomixer for 2 hours at 23 °C. The fluid phase was then separated and diluted with ten volumes of ice-cold water. The proteoliposomes were sedimented in a TLA120.2 rotor at 70,000 rpm for 30 minutes, and resuspended in 16.7 μL liposome resuspension buffer (100 mM KAc, 50 mM HEPES pH 7.4, 2 mM $Mg(Ac)_2$, 250 mM sucrose, 1 mM DTT). Substrates for insertions were prepared by translating in RRL for 15 min, then the reactions were treated with 1 mM puromycin to prevent further synthesis. Insertion reactions consisted of 8 μL translation in RRL, 1 mM EGTA, and 2 μL buffer, liposomes, proteoliposomes, or isolated mitochondria. The reactions were incubated at 32 °C for 30 min, unless indicated otherwise, before being treated with 0.5 mg/mL PK for 1 hour on ice. The reactions were quenched and the protected fragments enriched with NiNTA resin as described above.

In order to determine the percentage of MTCH2 that was inserted in the correct orientation, we reconstituted GFP-SUMO^{Eu1}-HA-MTCH2 into proteoliposomes as described above. We then mixed 1 μL of the final resuspension with SENP^{EuB} to a final concentration of 5 μM in the

presence or absence of 1% Triton X-100. Cleavage reactions were incubated on ice for 30 minutes and analyzed by immunoblotting with a MTCH2 antibody.

Flow cytometry

For all reporter experiments, respective K562 cell lines were spinfected with lenti-virus for indicated constructs and analyzed by flow cytometry after 48–72 hours. All reporter experiments were performed at least twice. For the apoptosis experiment, wild-type K562 cells expressing either MTCH2-P2A-BFP or BFP alone were treated with 5 μM imatinib mesylate (461080010, Fisher Scientific, USA) for 72 hours. Cells were harvested, washed once with ice cold PBS, then resuspended in 100 μL staining buffer (10 mM HEPES, 140 mM NaCl, 2.5 mM CaCl2 pH 7.4, 5% Annexin-FITC (Invitrogen, A13199), 50 μg/mL propidium iodide (P1304MP, Invitrogen, USA) before analysis by flow cytometry. For treatment with the GPAT inhibitor FSG67, K562 cells were treated for 16 hours with 75 μM FSG67 (Cedarlane Laboratories Cat. #10-4577) as previously described (*14*) prior to analysis by flow cytometry. All samples were run on either an NXT Flow Cytometer (Thermo Fisher) or a MACSQuant VYB (Miltenyi Biotec). Flow cytometry data was analyzed either in FlowJo v10.8 Software (BD Life Sciences) or Python using the FlowCytometryTools package.

Quantitative PCR

RNA levels were measured with quantitative PCR. Cells treated with a non-targeting guide or a guide targeting MTCH2 for eight days, and then RNA was extracted using an RNeasy kit (74104, Qiagen). The RNA was treated with amplification grade DNase I (18068015, Invitrogen), and then reversed transcribed using the SuperScript III First-Strand Synthesis SuperMix for qRT-PCR kit (11752050, Invitrogen), according to the manufacturer's instructions. The reactions were analyzed using a StepOnePlus Real-Time PCR system. The relative expression ratios were calculated compared to the housekeeping gene HPRT1, using primer efficiencies measured using a standard dilution curve. The data is in triplicate, and means and standard deviations are plotted. The primers used were as follows: MTCH2 (fwd GTCAGCTTCCTGGTCTCTTTAG, rev CCTTGTCACTCTCCTGGTAATG); OMP25 (fwd GTGCATATCCTCCATCCCATAG, rev GGTCTTACCGGACCTCTTATT); CYB5B (fwd ACAGCCAGGTGAAAGCTAAA, rev CCAACACCCTCTATTTCGGTAG); RHOT2 (fwd AGCGTCTACAAGCACCATTAC, rev AGGACCCTGTAGAGTGAGAAG); VDAC (fwd CAATACACTAGGCACCGAGATT, rev TCTGTCCCGTCATTCACATTAG); FUND1 (fwd GTAGGTGGTGGCTTTCTTCTT, rev CTGCTTTGTTCGCTCGTTTC); CYC1 (fwd CAAGTGGTCAGTCCTGAAGAG, rev CTTCCAGATGAAGAGAGGCTTAG).

Microscopy

To visualize localization to the mitochondria, K562 cells were stained with 1 μM Mitotracker Deep Red FM (M22426, Thermo Fisher) for 30 minutes. Cells were collected, spun down, washed in fresh media and plated onto a 96-well glass bottomed plate (160376, Thermo Fisher). K562 cells were then briefly spun down by centrifugation at 100*g* for 5 minutes and imaged on a Zeiss LSM710 NLO Laser Scanning confocal microscope.

Sequence alignments

An alignment of individual SLC25 repeats (Fig. 4A, S15) from various human transporter was generated using the hmmalign tool from the HMMER3.3 package (*61*) and a precomputed hmm profile of the SLC25 family from pfam (pf00153) (*62*). An alignment of MTCH2 homologues was generated using MUSCLE (*63*) and the ESPRIPT 3.0 server (https://espript.ibcp.fr, (*64*)) was used to display the alignment (fig. S16).

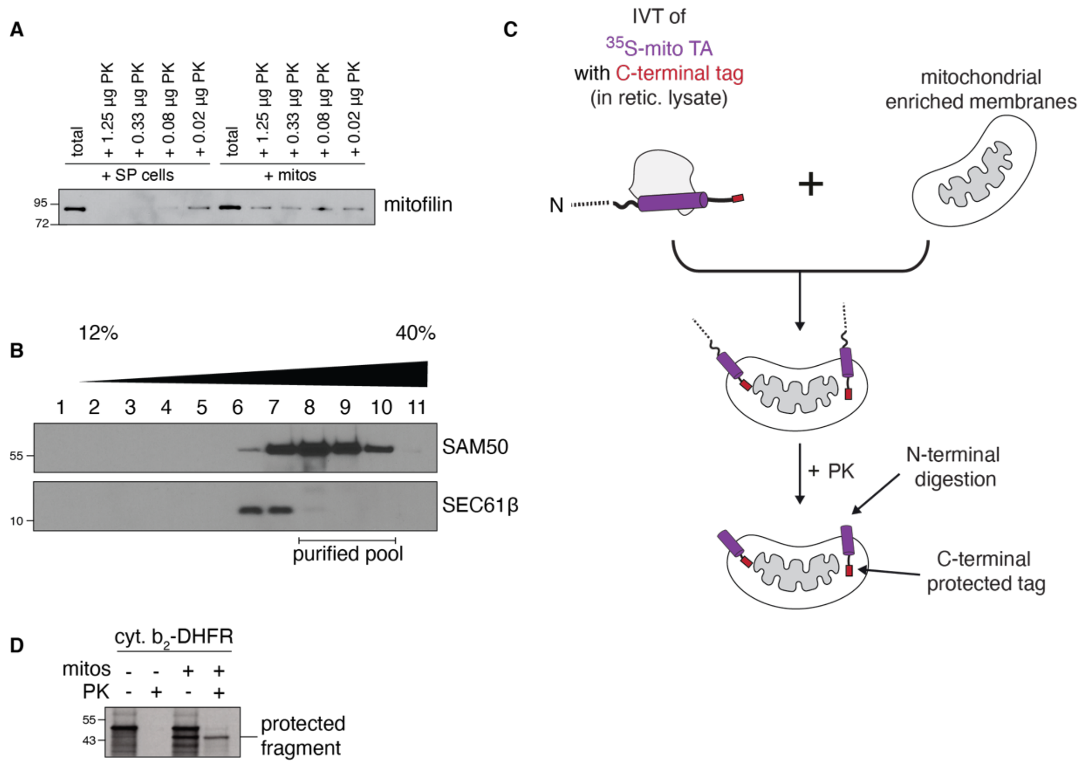

Fig. S1. In vitro protease protection assays into human mitochondria. **(A)** The integrity of the outer membrane was tested using both semi-permeabilized K562 cells and isolated mitochondria by treating each with decreasing amounts of proteinase K (PK). The resulting reactions were analyzed by immunoblot for the mitochondrial intermembrane space (IMS) protein mitofilin, which should be inaccessible to PK when the outer membrane is intact. **(B)** Further purification of mitochondria from K562 cells using a percoll gradient and density centrifugation. Blots for a mitochondrial marker (SAM50) and an ER marker (SEC61β) demonstrate relative proportion of mitochondria and ER in the differential-centrifugation isolated mitochondria that are used for most in vitro experiments. Proteomics and crosslinking-mass spectrometry experiments were performed with percoll-gradient enriched mitochondria and used fractions 8-10 (Fig. 2A, Fig. 3D). **(C)** Schematic of the in vitro insertion assay, using protease protection of a C-terminal appended tag as a readout for insertion. In all insertion assays, substrates were translated using an in vitro translation reaction (IVT) in rabbit reticulocyte lysate supplemented with ^{35}S-methionine, permitting detection by autoradiography. Translation was terminated by addition of puromycin, followed by incubation with isolated mitochondria derived from human K562 cells. After incubating with mitochondria, reactions were treated with PK. For TA proteins, the resulting protease protected band was immunoprecipitated via a tag on its C-terminus, ensuring insertion into the outer membrane in the correct orientation, and analyzed by SDS-PAGE and autoradiography. **(D)** The IMS-localized TOM substrate composed of the cytochrome b targeting sequence fused to the inert protein DHFR (cyt. b_2-DHFR) was translated and incubated with isolated mitochondria to confirm their activity. Insertion was detected by protease protection as described.

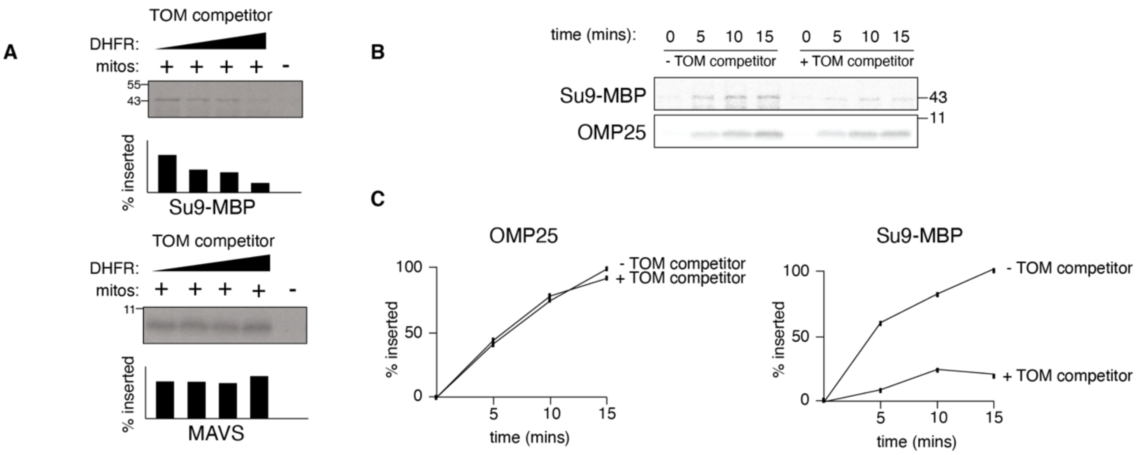

Fig. S2. Insertion of mitochondrial TA proteins is not strictly dependent on the TOM40 translocase. (A) Competition assay as described in Fig. 1A with the mitochondrial TA protein, MAVS. Here, a fixed amount of mitochondria is incubated with increasing concentrations of purified, unlabeled Su9-DHFR (SU9-fused to the insert sequence DHFR; referred to as DHFR in the figure), a canonical TOM40 dependent substrate, and radiolabeled Su9-MBP or MAVS. As shown, there is a Su9-DHFR concentration dependent decrease in Su9-MBP insertion, assessed using the protease protection assay described in fig. S1C. This indicates that Su9-MBP relies on the same TOM40 mediated pathway for its insertion as Su9-DHFR. By contrast, MAVS, a mitochondrial outer membrane TA protein, inserts similarly into mitochondria independent of how much Su9-DHFR is present in the insertion reaction. This suggests that MAVS can utilize a TOM40 independent route for insertion into the outer membrane. **(B)** As in Fig. 1A, ^{35}S-methionine labelled TOM substrate Su9-MBP or the mitochondrial TA protein OMP25 were translated in rabbit reticulocyte lysate, released from the ribosome using puromycin and incubated with purified mitochondria either in the absence or presence of the recombinant TOM competitor Su9-DHFR (at a fixed concentration). Mitochondrial insertion was assessed at five minute intervals by protease protection, SDS-PAGE and autoradiography. **(C)** Time course of insertion from (B) quantified. Error bars represent one standard deviation from three independent biological replicates.

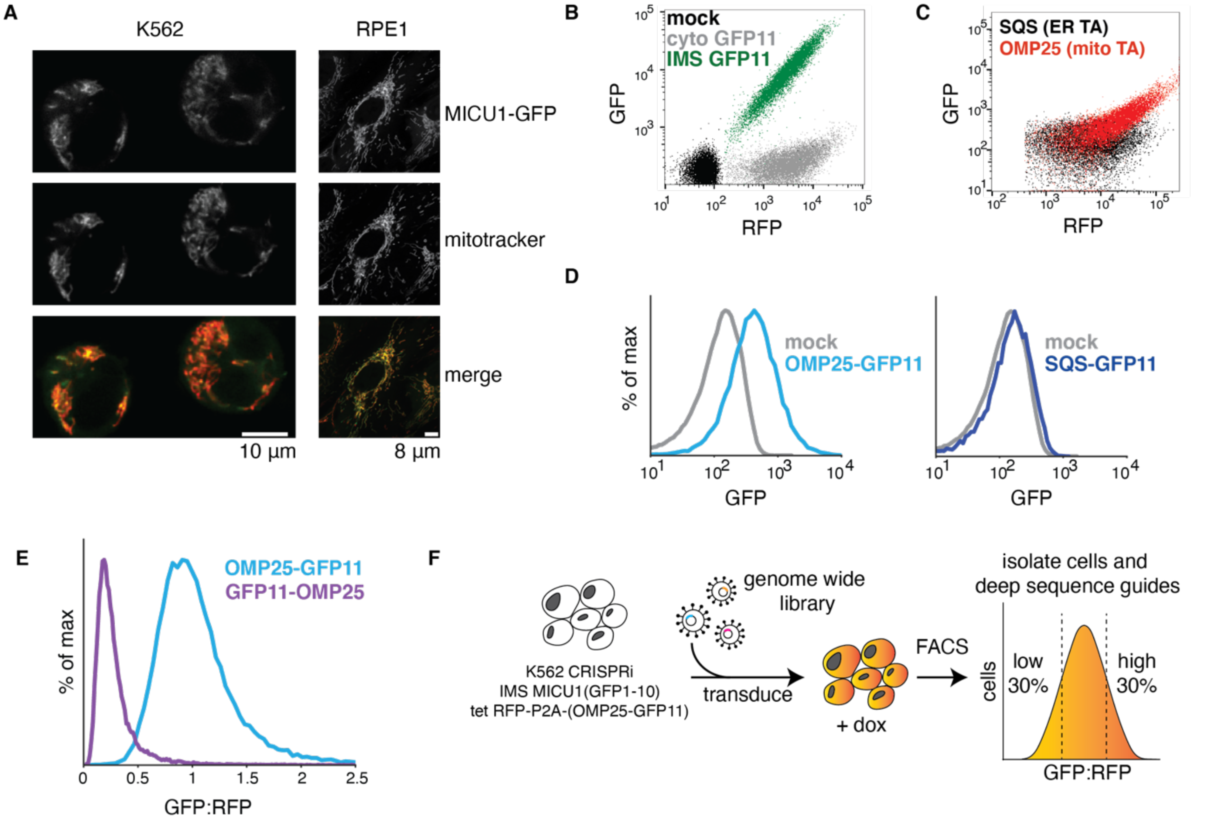

Fig. S3. A CRISPRi screening platform to identify factors required for mitochondrial TA biogenesis in human cells. **(A)** Microscopy showing that the IMS-targeting sequence from MICU1 conjugated to full length GFP results in its localization to the mitochondria in mammalian K562 and RPE1 CRISPRi cells. **(B)** Either a cytosolic GFP11 or IMS targeted MICU-GFP11 were inserted in the backbone containing a translational control (RFP) separated by a viral 2A sequence (see Fig. 1B). These were independently expressed in K562 CRISPRi cells stably expressing MICU-GFP1-10 and analyzed by flow cytometry. Cytosolic GFP11 negligibly conjugated with IMS targeted GFP1-10, suggesting its correct localization to the IMS. **(C)** The endogenous sequences of two TA proteins: SQS, which is localized to the ER, and OMP25, which under these conditions is dual-localized to both the outer membrane and ER, were appended to a C-terminal GFP11 in the expression cassette described in Figure 1B. These constructs were independently introduced into cells expressing IMS localized GFP1-10 and analyzed by flow cytometry. **(D)** Histograms of (B) comparing GFP fluorescence for OMP25 and SQS compared to a mock transduced control. The marked increase in GFP fluorescence suggest that OMP25, but not SQS can successfully conjugate with GFP1-10 localized to the IMS. **(E)** OMP25 conjugated to either an N- or C-terminal GFP11 were expressed in the expression cassette described in Fig. 1B in cells expressing IMS localized GFP1-10 and analyzed by flow cytometry. The majority of OMP25 is correctly integrated in an N-cytosolic, C-IMS orientation. **(F)** Workflow of the FACS-based CRISPRi screen. A K562 CRISPRi reporter cell line was constructed that constitutively expressed GFP1-10 in the IMS and the OMP25-GFP11 reporter under an inducible promoter. For the screen, these cells were transduced with a genome-

scale CRISPR interference (CRISPRi) sgRNA library and then the OMP25-GFP11 reporter was induced with doxycycline for 24 hours prior to cell sorting. Cells were sorted based on ratiometric changes in GFP relative to RFP, and sgRNAs expressed in the isolated cells were identified using deep sequencing.

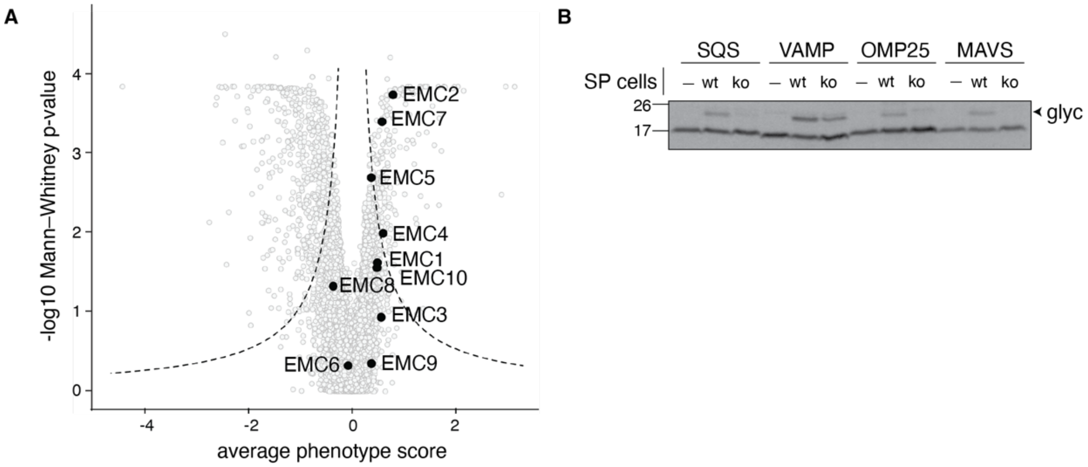

Fig. S4. The ER membrane protein complex (EMC) is required for insertion of mislocalized mitochondrial TAs to the ER. **(A)** Volcano plot of the genome-wide CRISPRi screen with EMC subunits shown in black. **(B)** A panel of ER (SQS and VAMP) and mitochondrial (OMP25 and MAVS) TA proteins were conjugated to a C-terminal opsin epitope. The opsin epitope contains a consensus glycosylation sequence that is modified upon insertion into the ER lumen. These constructs were then translated in rabbit reticulocyte lysate in the presence of ^{35}S-methionine. The reactions were puromycin treated and incubated with either wild-type (wt) or EMC knockout (ko) semi-permeabilized (SP) cells. Insertion into the ER, as monitored by appearance of a glycosylated band ('glyc'), was dependent on the EMC for its canonical substrate SQS, and both mitochondrial TAs. In contrast, VAMP's insertion was unaffected by EMC knockout, consistent with its previously reported dependence on the GET pathway for insertion (*38*).

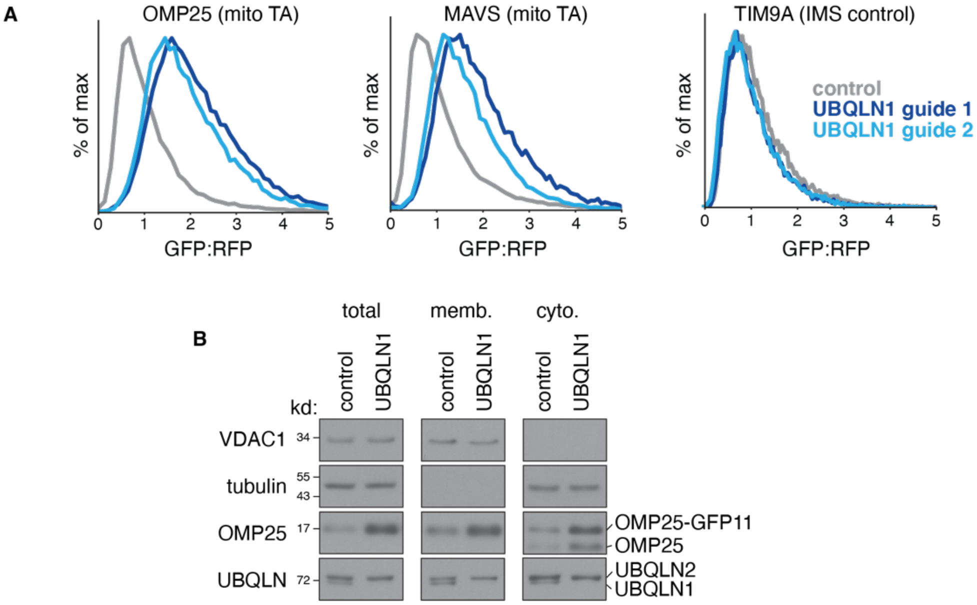

Fig. S5. The UBQLNs are quality control factors for mitochondrial TAs. **(A)** K562 CRISPRi cells expressing IMS GFP1-10 were depleted of UBQLN1 using two different sgRNAs. Reporters were introduced for either two mitochondrial TAs (OMP25 and MAVS) or an IMS localized control (TIM9A) and cells were analyzed by flow cytometry. Lack of UBQLN1 results in a ratiometric increase in GFP:RFP fluorescence for mitochondrial TAs, consistent with its previously reported role in targeting mislocalized mitochondrial TAs for degradation by the ubiquitin proteasome pathway (*12*). **(B)** K562 CRISPRi cells expressing IMS GFP1-10 and OMP25-GFP11 under an inducible promoter were depleted of UBQLN1 using sgRNA (above, guide 1). After doxycycline induced expression of OMP25-GFP11, cells were lysed and fractionated into cytosolic and membrane fractions using centrifugation. Levels of the indicated proteins were then assessed by SDS-PAGE and immunoblotting. In the absence of UBQLN1, more endogenous and GFP11 tagged OMP25 were found in both the cytosolic and membrane fractions.

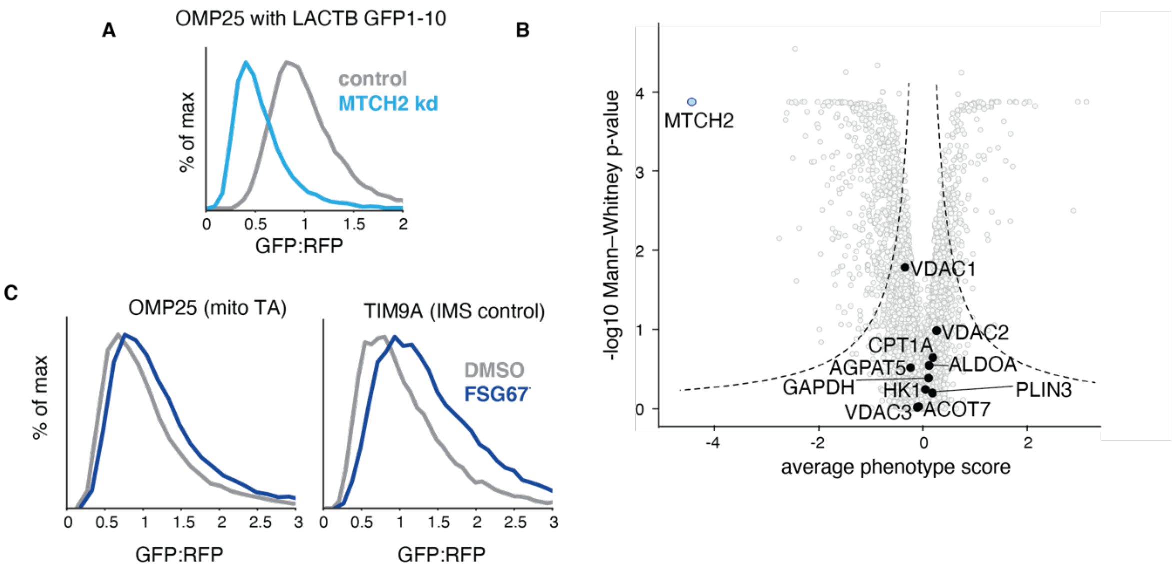

Fig. S6. **Assessing the effects of lipid biogenesis defects on mitochondrial TAs. (A)** Flow cytometry analysis as in Fig. 1D but with an alternative IMS targeting sequence derived from LACTB (*41*) appended to the GFP1-10. (**B**) Volcano plot of the genome-wide CRISPRi screen indicating MTCH2 (in light blue) and factors previously implicated as mediators of MTCH2-dependent outer membrane fatty acid synthesis or transport (in black). **(C)** K562 IMS GFP1-10 expressing cells were treated with the pan Glycerol 3-phosphate acyltransferase (GPAT) inhibitor FSG67 for 16 hours (75 µM) or a vehicle. MTCH2-dependent mitochondrial fusion has been shown to require GPAT catalyzed LPA synthesis. A reporter expressing either a mitochondrial TA (OMP25) or an IMS localized control (TIM9A) were expressed in GPATi treated cells and analyzed by flow cytometry.

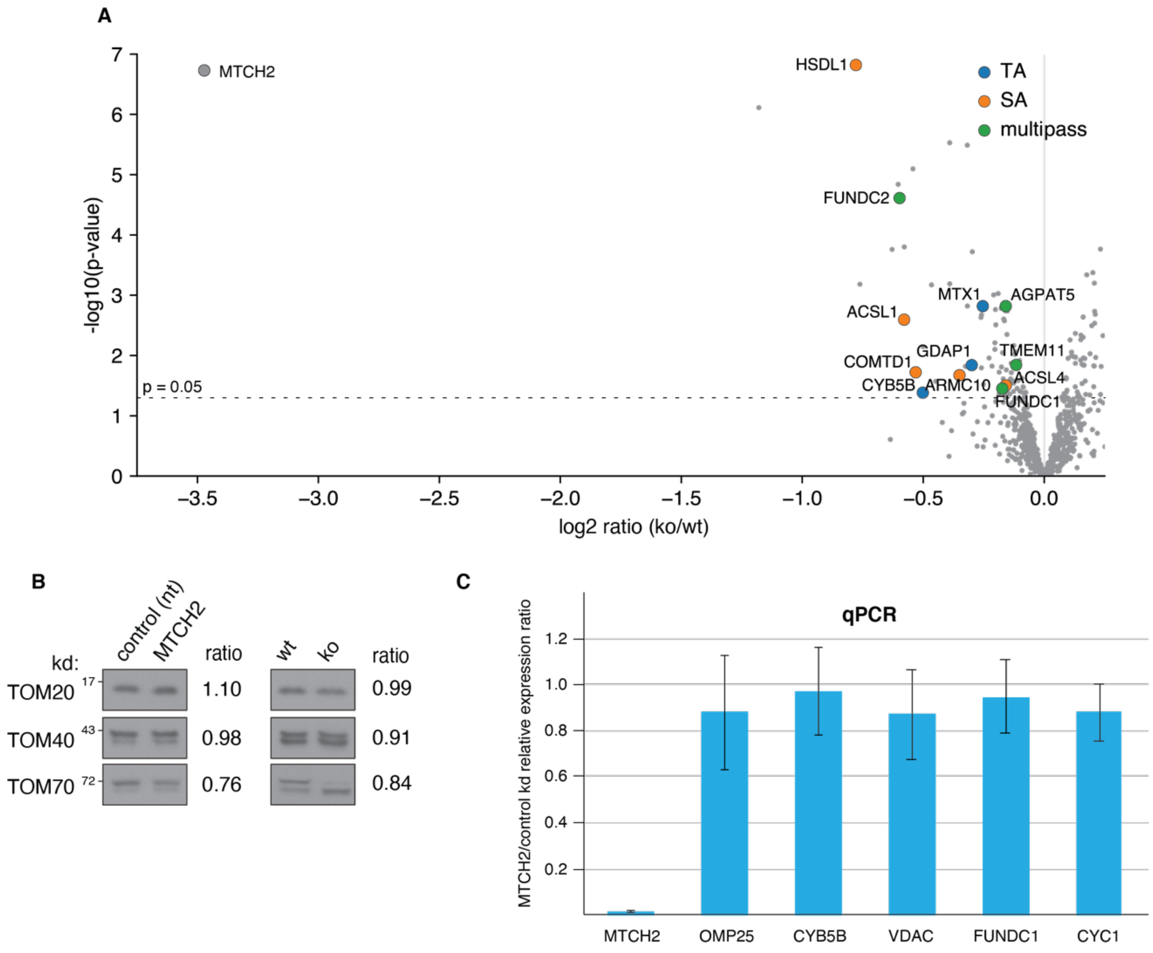

Fig. S7. Analysis of proteomic and transcriptomic changes to mitochondrial outer membrane proteins in MTCH2 depleted cells (**A**) Label-free mass spectrometry analysis as in Fig. 2A of crude mitochondria isolated from K562 cells via differential centrifugation with MTCH2 knockout (ko) versus wild-type (wt) cells. Highlighted are select mitochondrial proteins that across four biological replicates were statistically altered by loss of MTCH2. (**B**) Immunoblotting of TOM complex subunits from MTCH2 depleted (kd) and non-targeting control cells (left), and wild-type and MTCH2 knockout (ko) cells (right) as in Fig. 2B. Quantification of fold-change in depleted vs control cells is displayed as determined using a dilution series for each antibody. (**C**) qPCR of mitochondrial genes in MTCH2 versus control knockdown in K562 cells (normalized to the housekeeping gene HPRT). See methods for specific details.

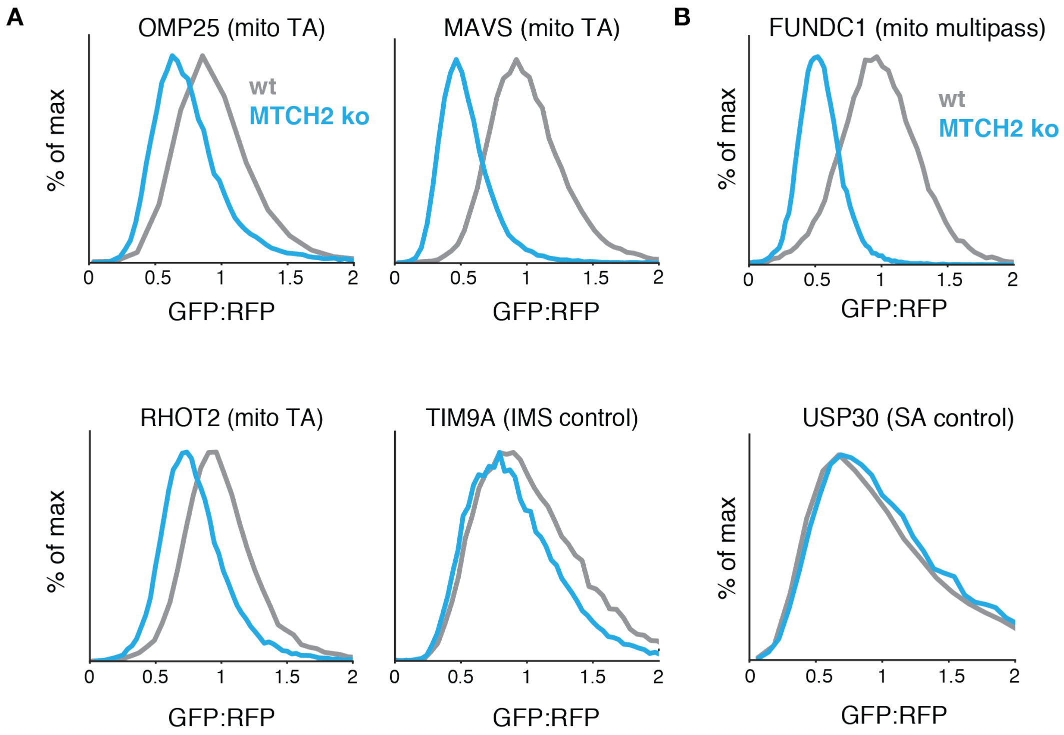

Fig. S8. **Analysis of TA proteins for MTCH2 dependent biogenesis in knockout cells.** As in Fig. 2C but in wild-type compared to MTCH2 knockout cells. Histograms summarizing flow cytometry analysis of the integration of the indicated mitochondrial proteins.

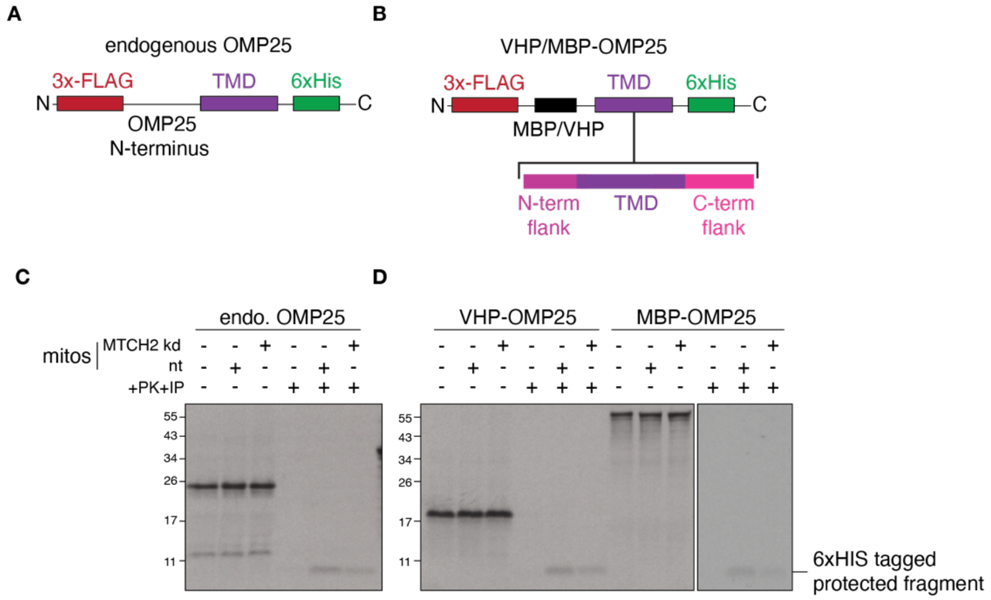

Fig. S9. Establishing an in vitro system to test differential dependence on MTCH2 on insertion of a panel of mitochondrial TAs. (A, B) Schematic of OMP25 constructs used to test whether the dependence on MTCH2 for insertion that we observed with the full-length endogenous OMP25 (A) could be recapitulated with an artificial N-terminus (B). **(C)** Using the protease protection assay described in fig. S1, we compared insertion of the endogenous OMP25 containing a C-terminal 6xHIS tag into mitochondria isolated from K562 cells expressing either a non-targeting (nt) or MTCH2 sgRNA (kd). We observed that loss of MTCH2 specifically decreased the levels of a protease protected fragment consistent in size with the TMD and C-terminus of OMP25 (compare lanes 5 vs 6). In the absence of mitochondria, no protected fragment is observed (lane 4). Because this protease protected fragment could be immunoprecipitated using a C-terminal 6xHIS tag, we verified that OMP25 was inserted in the correct orientation, with its C-terminus in the IMS and its N-terminus facing the cytosol. **(D)** As in (A) but using a fusion of the OMP25 TMD and its flanking residues to both the unrelated globular proteins MBP and VHP. We concluded that the OMP25 TMD alone is sufficient to confer MTCH2 dependent insertion on both of the tested fusion proteins (compare lanes 5 vs 6 and 11 vs 12). Because the VHP-fusions were translated more efficiently, we generated a panel of mitochondrial TAs using the depicted VHP N-terminus as shown in (B). These were used for experiments in Fig. 3B, C, fig. S10-11.

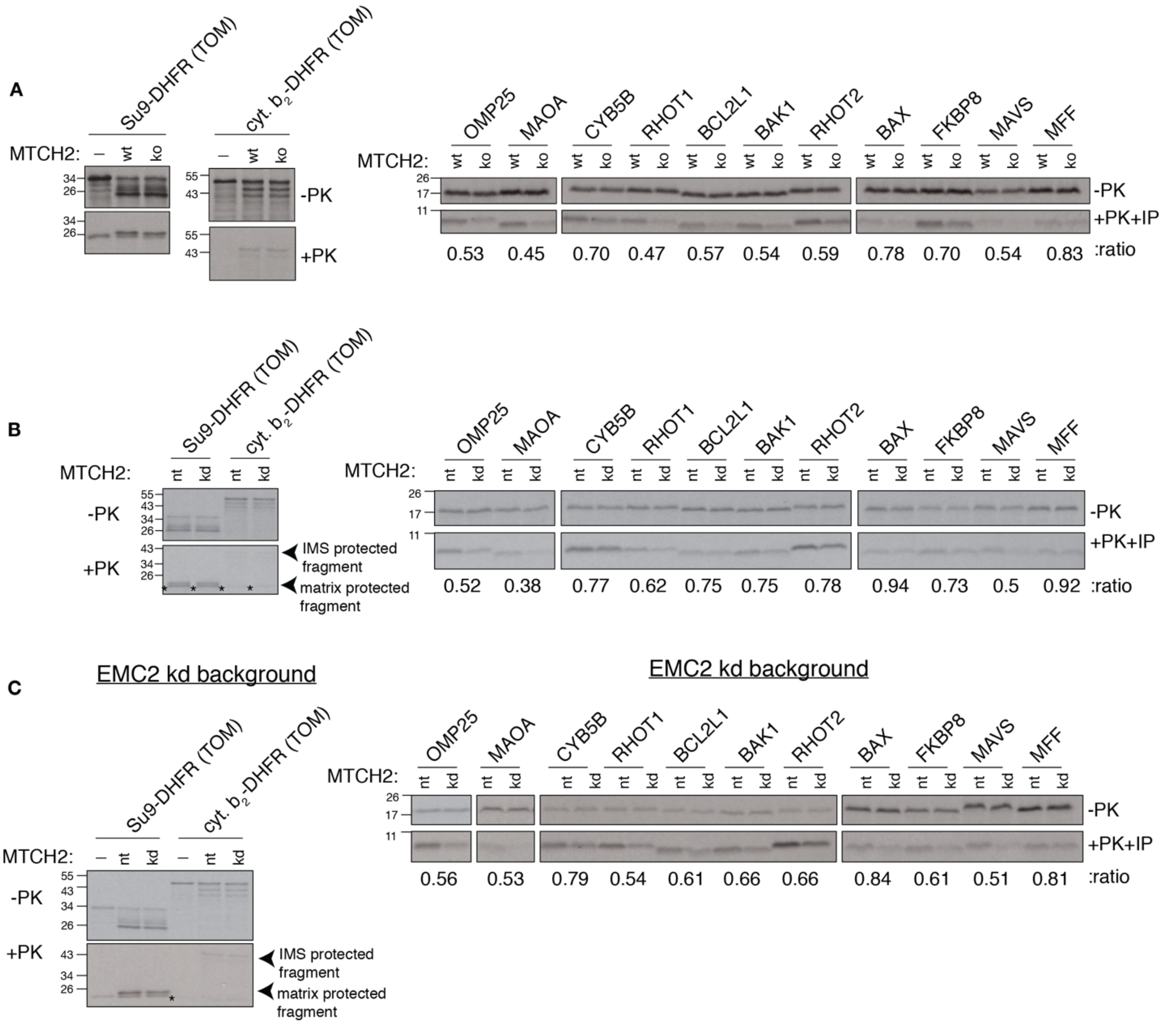

Fig. S10. Insertion of mitochondrial TAs in vitro is affected by MTCH2 knockdown. (A) ^{35}S-methionine labelled TA proteins were analyzed for in vitro insertion into mitochondria isolated from wild-type (wt) or MTCH2 knockout (ko) K562 cells. Here, the indicated TMDs and flanking residues were fused to VHP as described in fig S9. Displayed are the samples prior to addition of protease (-PK) and the protease protected fragment that has been affinity purified via a 6xHIS tag on the C-terminus of each substrate (PK+IP), ensuring insertion in the correct topology. Canonical TOM substrate controls that are targeted to either the matrix (Su9-DHFR) or IMS (cyt. b_2-DHFR) were tested in parallel. Relative insertion efficiency in MTCH2 depleted/wild-type mitochondria has been quantitated and displayed for each substrate. **(B)** As in A except using mitochondria isolated from K562 CRISPRi cells expressing either a non-targeting (nt) or MTCH2 targeting (kd) sgRNA. A panel of mitochondrial TAs was tested in parallel with a matrix (Su9-DHFR) and IMS (cyt. b_2-DHFR) targeted control that rely on the TOM pathway, which were unaffected**.** *Denotes the folded, protease resistant, DHFR domain that migrates immediately below the mature, matrix targeted control, and is visible in the absence of mitochondria. **(C)** Note that because some residual ER is present in the enriched

mitochondrial membranes used in these insertion reactions (see fig. S1), for those substrates that are dual localized, we cannot formally differentiate between insertion into mitochondria vs ER in this assay alone. To address this, we have tested the complete TA panel in an EMC knockdown background, which we found eliminated mistargeting of mitochondrial TAs to the ER (fig. S4). Therefore, we perform insertion assays as in (A) except using mitochondria isolated from either EMC2 knockdown or EMC2 and MTCH2 knockdown K562 CRISPRi cells.

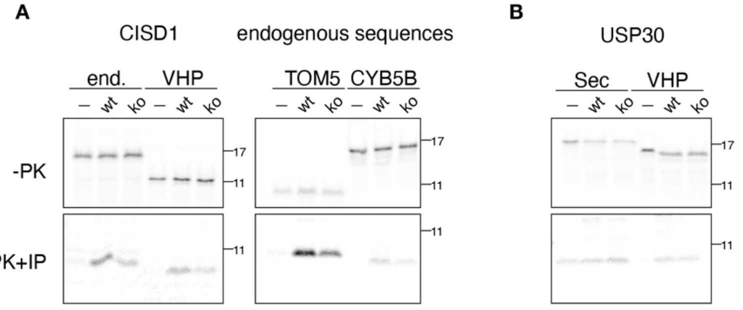

Fig. S11. Insertion of α-helical outer membrane proteins display MTCH2 dependence in concordance with in vivo experiments. (A) The indicated ^{35}S-methionine labeled TA or signal anchored proteins were translated in rabbit reticulocyte lysate and following treatment with puromycin were incubated with wt or MTCH2 ko mitochondria. Successful insertion was determined by protease treatment and immunoprecipitation of the protected C-terminal 6xHIS tagged fragment. We find that insertion of the signal anchored protein CISD1, either when using the endogenous sequence or when its TMD is fused to the VHP cassette (described in fig. S9) is MTCH2 dependent. Similarly, endogenous TOM5 requires MTCH2 for insertion in vitro, consistent with our in vivo florescent reporter data (Fig. 2D). **(B)** To test insertion of the signal anchored protein USP30, we generated constructs in which the USP30 TMD and flanking residues was fused to an inert C-terminal linker along with two affinity tags on either termini (6xHis-USP30 TMD-sec/VHP-3xFLAG). We found that insertion of both of these USP30-fusion proteins is not affected by loss of MTCH2, consistent with our in vivo data (Fig. 1E). The USP30 TMD alone is sufficient to confer MTCH2 independent insertion, as insertion of fusions of the USP30 TMD to either the N-terminus of SEC61β or the inert globular protein VHP are both MTCH2 independent.

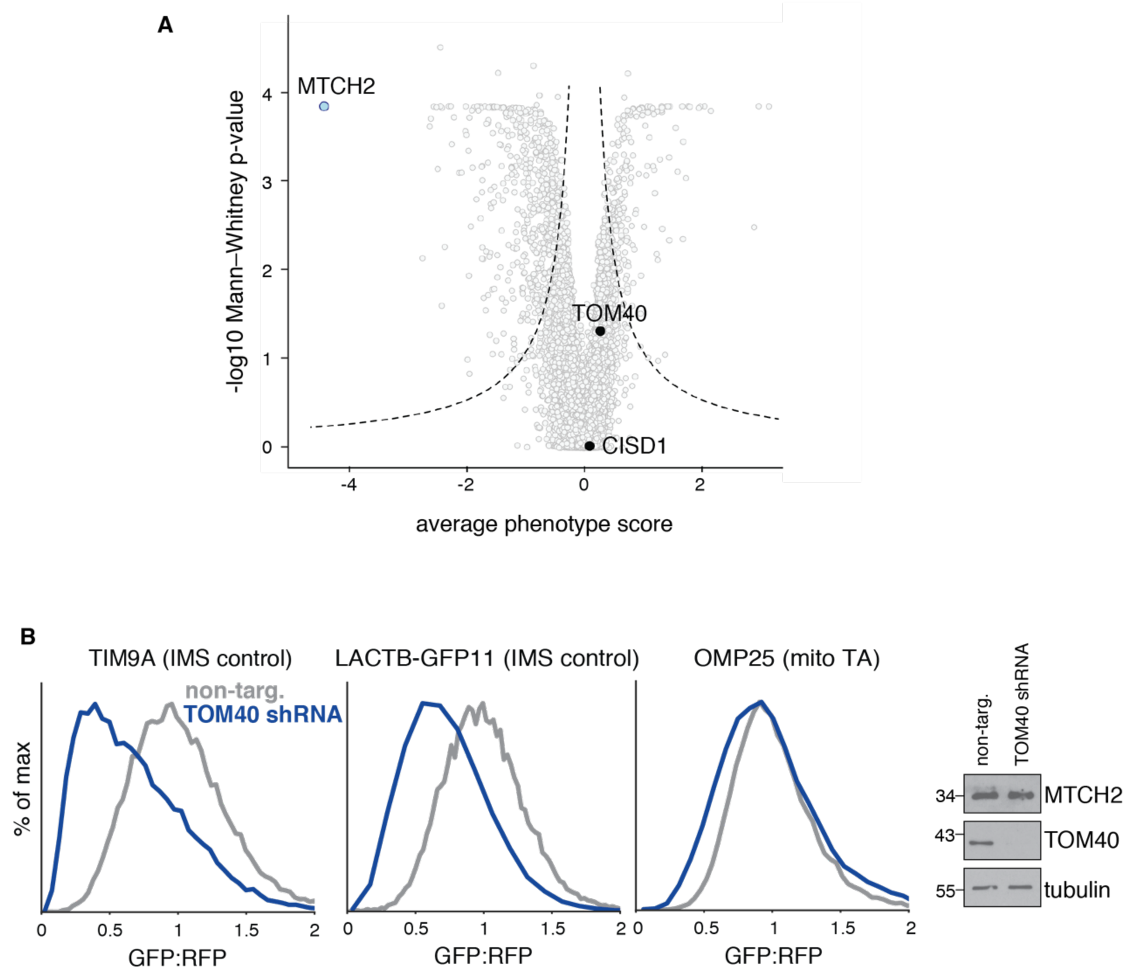

Fig. S12. TOM40 and CISD1 are not strictly required for mitochondrial TA biogenesis. (A) Volcano plot of the genome-wide CRISPRi screen as in Fig. 1C indicating MTCH2 (in light blue) and the two other factors (TOM40 and CISD1, in black) that were enriched upon crosslinking OMP25 with purified mitochondria (Fig. 3D; see fig. S1C for mitochondrial purification). **(B)** Using our fluorescent reporter assay (Fig. 1B) we show that depletion of TOM40 using an shRNA (dark blue) compared to a scrambled control (grey) in wild-type cells (wt) does not affect the biogenesis of the mitochondrial TA OMP25 under conditions where biogenesis of the canonical TOM substrates LACTB and TIM9A is diminished. These results are consistent with the TOM40 competition experiment (Fig. 1A, fig. S2) that shows that mitochondrial TA insertion in vitro is not affected by addition of a recombinant TOM40 substrate. Together these data suggest that mitochondrial TAs do not strictly require TOM40 for their biogenesis. However, we do not formally exclude a potential contribution of the TOM complex to either the recruitment or insertion of alpha-helical proteins into the outer membrane.

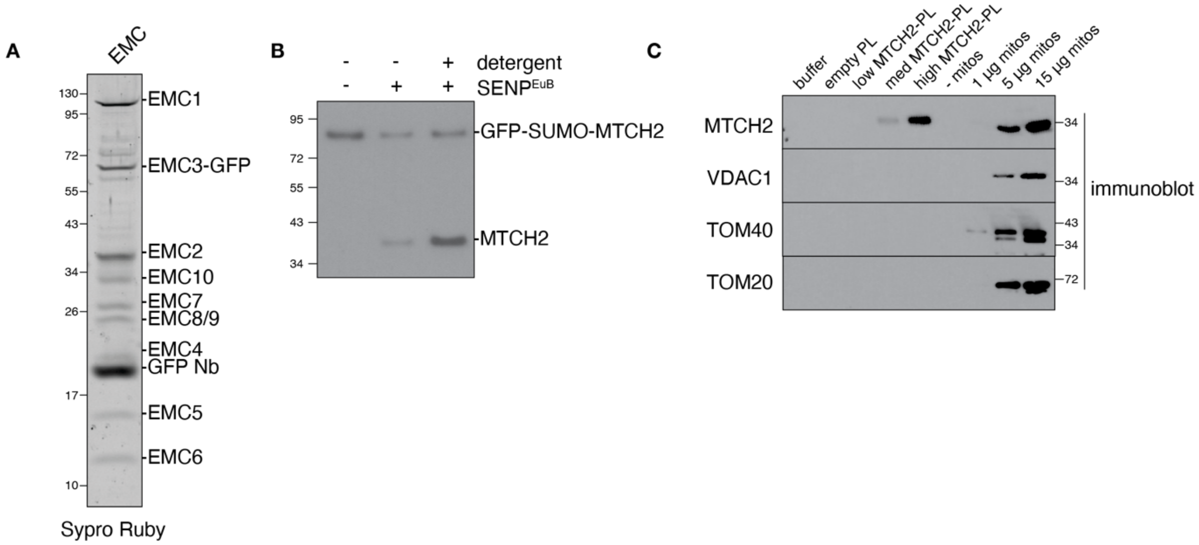

Fig. S13. Establishing a reconstituted system to query MTCH2-dependent insertion into proteoliposomes. (A) The human EMC was expressed and purified as previously described using a GFP fused to the C-terminus of EMC3 in the detergent DBC (*24*) and visualized using Sypro Ruby staining. This was further used for generating EMC-proteoliposomes used in insertion reactions shown in fig. S14. **(B)** Following reconstitution of purified MTCH2 into proteoliposomes at lipid:protein ratios ranging from 6000:1 (low MTCH2) to 1000:1 (high MTCH2), we sought to determine the fraction that was correctly oriented relative to the cytosol (i.e. with the N-terminus facing the cytosol and the C-terminus within the lumen of the liposome). Because we found that MTCH2 is largely protease resistant (Fig. 3H), we could not use the classical strategy to determine its orientation after reconstitution. Therefore, we expressed and purified an N-terminally tagged GFP-SUMO-HA-MTCH2, such that the GFP tag and SUMO protease cleavage site would be exposed to the cytosol upon reconstitution in the correct orientation. Following reconstitution of GFP-SUMO-HA-MTCH2, intact proteoliposomes or matched controls that were solubilized by addition of 1% Triton, were treated with SUMO protease (SENP-EuB). Using this strategy, we used immunoblotting to determine that ~50% of GFP-SUMO-HA-MTCH2 is oriented correctly following reconstitution. **(C)** Analysis of the contents of reconstituted MTCH2 proteoliposomes as determined by immunoblotting for the indicated proteins. These results suggests that the TOM complex subunits, as well as the abundant outer membrane β-barrel protein, VDAC, are not present at appreciable amounts in the purified proteoliposomes. Therefore, trace levels of the TOM complex are unlikely to be strictly required for any observed insertion activity of purified, reconstituted MTCH2.

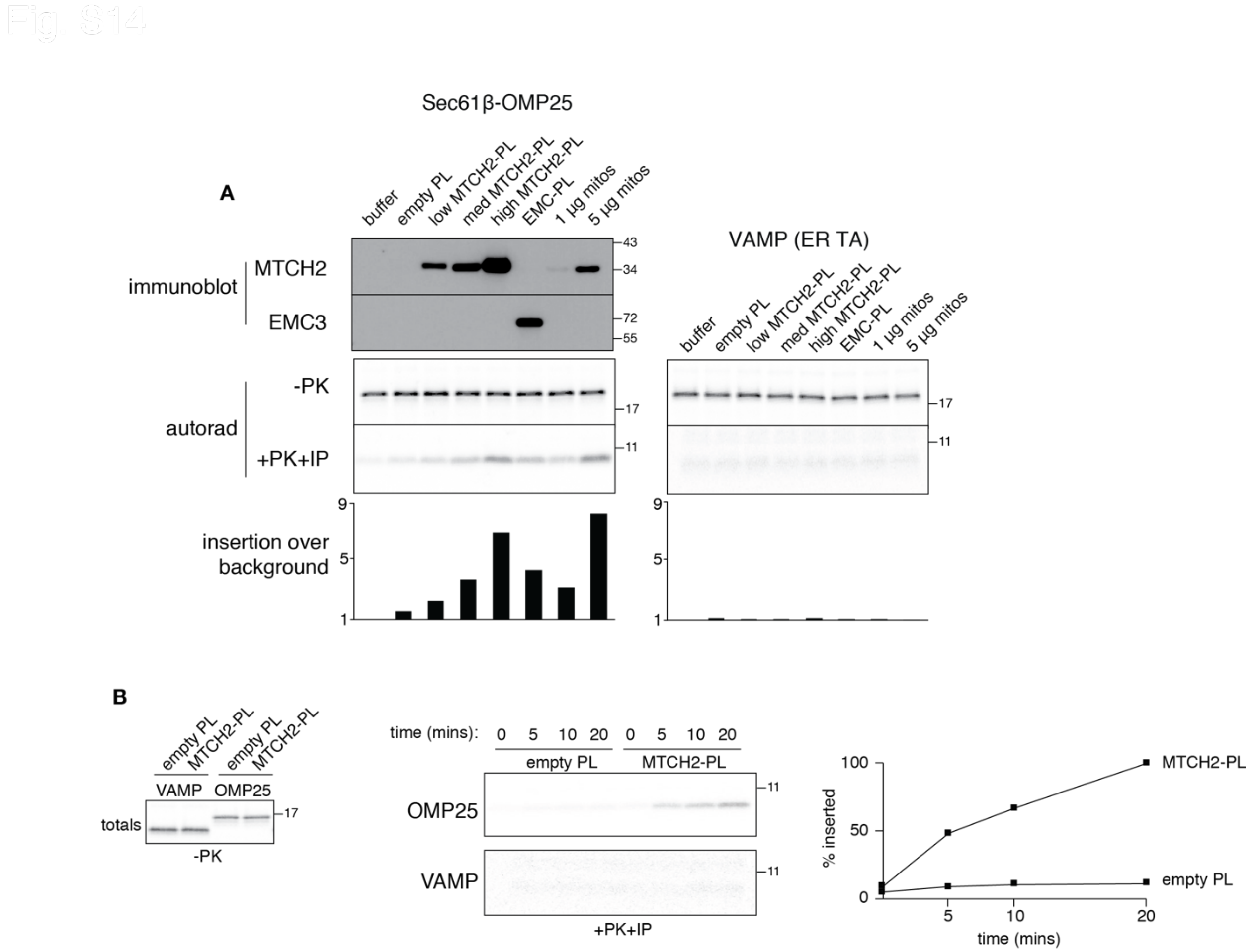

Fig. S14. MTCH2 is sufficient to stimulate insertion of mitochondrial TAs into proteoliposomes at an efficiency similar to purified mitochondria and the EMC. (A) Following reconstitution, the recovered proteoliposomes were analyzed by immunoblotting for incorporation of the indicated proteins in comparison to purified mitochondria. Using a protease protection assay, Sec61β-OMP25 or the ER-TA, VAMP (a GET pathway substrate), synthesized in rabbit reticulocyte lysate was tested for insertion into liposomes reconstituted with increasing amounts of purified MTCH2, the human EMC, or an empty control. In parallel for comparison, we tested insertion into mitochondria isolated from K562s at two concentrations. The resulting protease protected fragments were immunoprecipitated, imaged by autoradiography (autorad), and their intensity quantified on a phosphoimager, and are displayed normalized to the cytosol only control. The presence of a small amount of protected fragment in cytosol alone may be indicative of chaperone binding. **(B)** As in (A), reconstituted proteoliposomes of MTCH2 or an empty control were incubated with OMP25 and VAMP for the indicated time points and the % inserted was quantitated using protease protection.

A

alignment		% ID
SLC25A46	MTCH1	16.5
SLC25A46	MTCH2	10.7
MTCH1	MTCH2	36.4

B

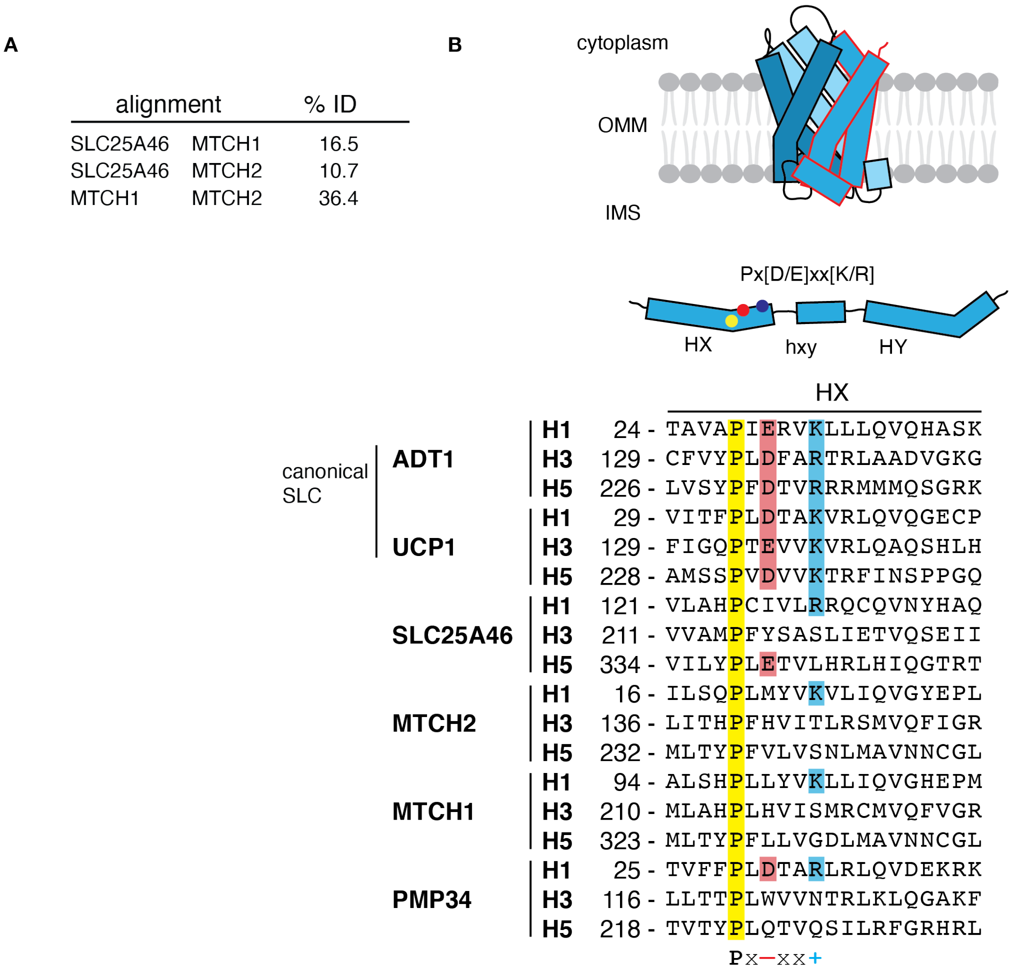

Fig. S15. Shared features of outer membrane SLC25 transporters. **(A)** Sequence identity derived from pairwise alignment between SLC25A46, MTCH1, and MTCH2. **(B)** On top, a cartoon representation of SLC25 TMD arrangement showing the three SLC25 repeats in unique shades of blue, with a single repeat outlined in red. In the middle, a schematic showing the location of characteristic motifs within a single SLC25 repeat, which normally encodes two TM helices. On bottom, sequence alignment of all individual SLC25 repeats from two inner membrane SLC25 transporters (ADT1, UCP1) and four outer membrane SLC25 transporters, three mitochondrial (SLC25A46, MTCH1, MTCH2) and one peroxisomal (PMP34), with residues from the Px[D/E]xx[K/R] motif highlighted.

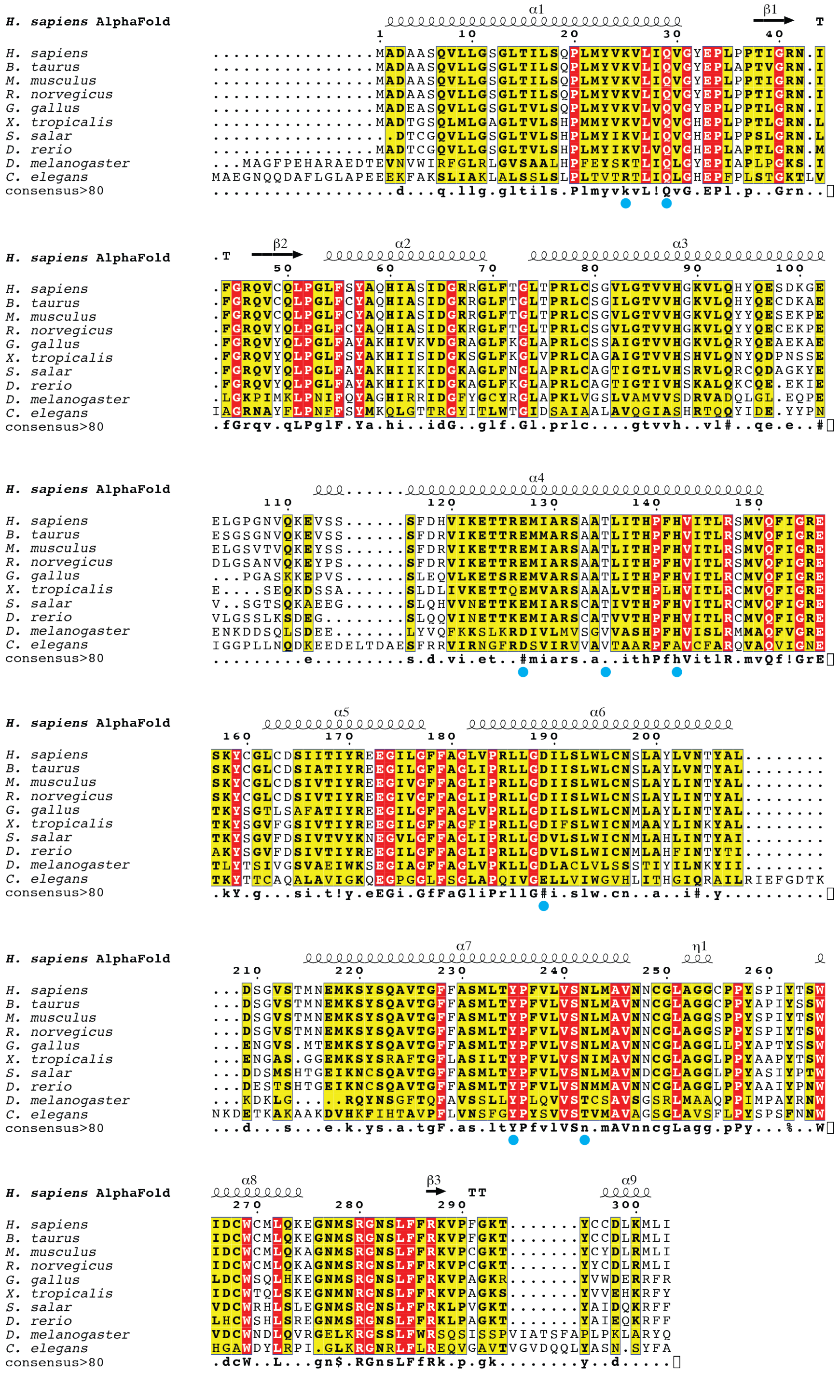

Fig. S16. Multisequence alignment of homologs of MTCH2.
MUSCLE (*63*) generated sequence alignment of MTCH2 homologues from a variety of animal species, generated using the ESPRIPT 3.0 server (https://espript.ibcp.fr, (*64*)). Red columns denote 100% sequence conservation, yellow columns denote moderate conservations with similar residues bolded. Secondary structure derived from the human MTCH2 Alphafold2-predicted model is displayed above the sequences

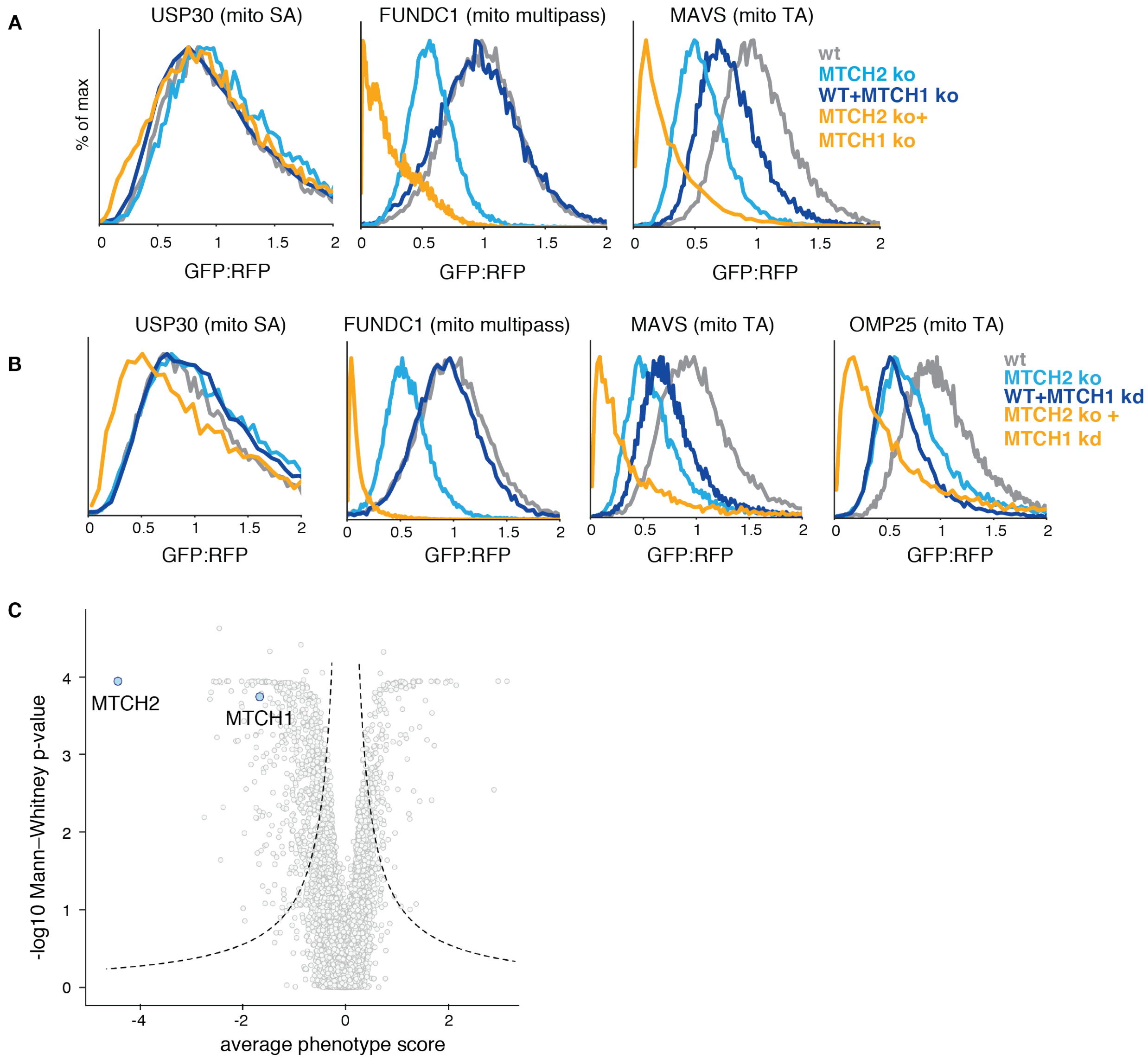

Fig. S17. MTCH1 acts in a parallel, and partially redundant, pathway to mediate insertion of mitochondrial TAs. (A) As in Fig. 4B for a set of outer membrane reporters including a signal-anchored protein (USP30), a mitochondrial TA (MAVS) and a multipass protein (FUNDC1). **(B)** As in (A) but in either wild-type or MTCH2 knockout (ko) K562 CRISPRi cells expressing guides targeting MTCH1 for knockdown. **(C)** Volcano plot of the genome-wide CRISPRi screen with MTCH1 and MTCH2 highlighted.

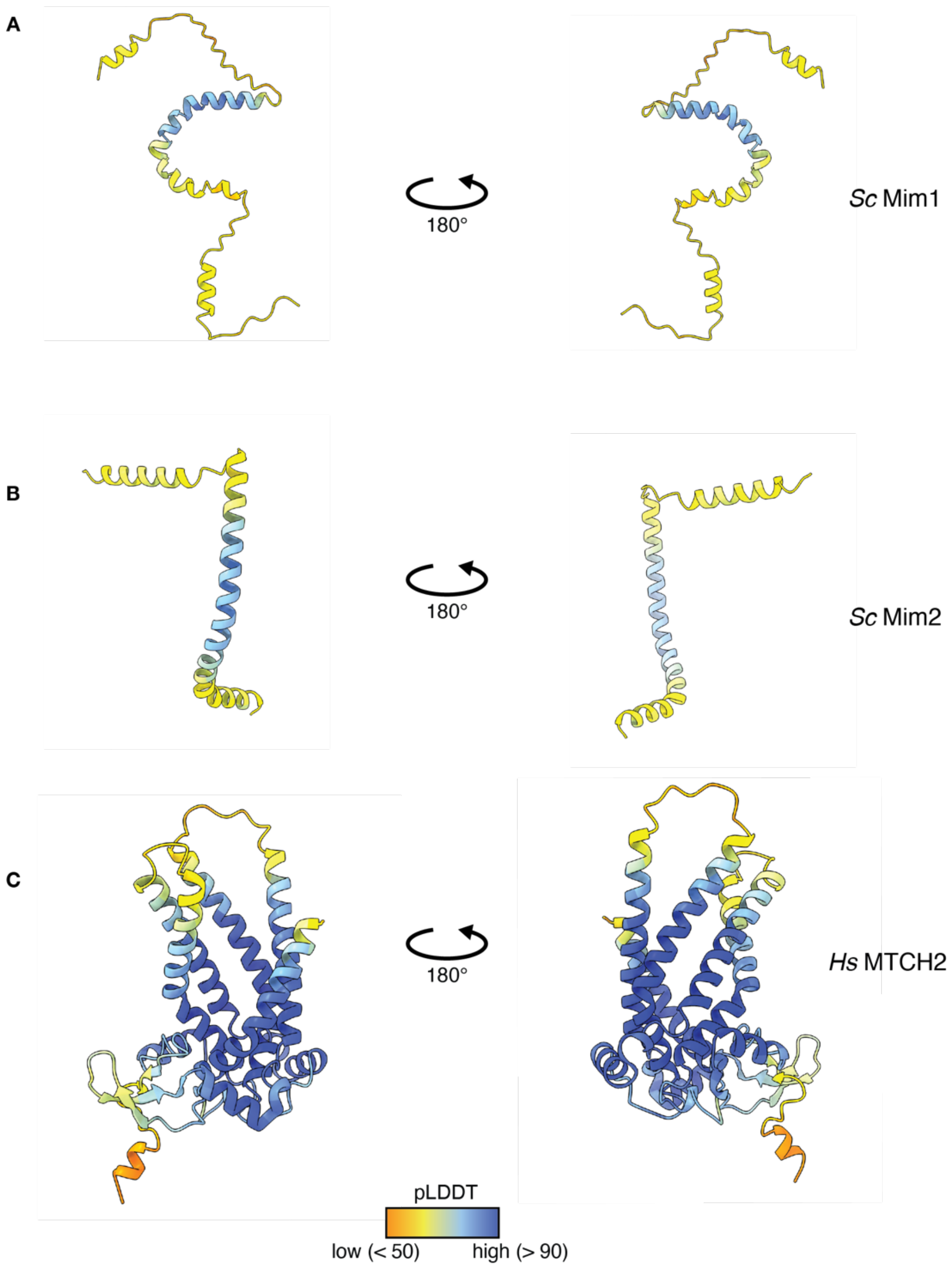

Fig. S18. Comparison of the AlphaFold2 predicted structures of the *S. cerevisiae* Mim1 and 2 with human MTCH2. Alphafold2 predicted models of the two subunits of the MIM complex from *S. cerevisiae*, Mim1 and Mim2. Neither bear significant resemblance to MTCH2 both at the sequence or topological level. Mim1 and Mim2 are 13 and 10 kDa proteins respectively, each containing a single transmembrane helix spanning the mitochondrial outer membrane. The MIM complex is estimated to be 150 kDa (via native PAGE analysis), presumably formed by oligimerization of multiple copies of each subunit (*65*). The MIM complex has been shown to be required for the insertion of many tail-anchored, signal-anchored, and multipass mitochondrial

outer membrane proteins in yeast (*6*, *66–69*). The extensive prior characterization of the MIM complex, as well as the outer membrane insertase pATOM36 from trypanosomes (*7*, *70*), is important for demonstrating the necessity of proteinaceous machinery in the insertion of transmembrane helices into the mitochondrial outer membrane. MTCH2 presumably evolved to perform a similar function via a completely unrelated protein fold, representing a striking case of convergent evolution.

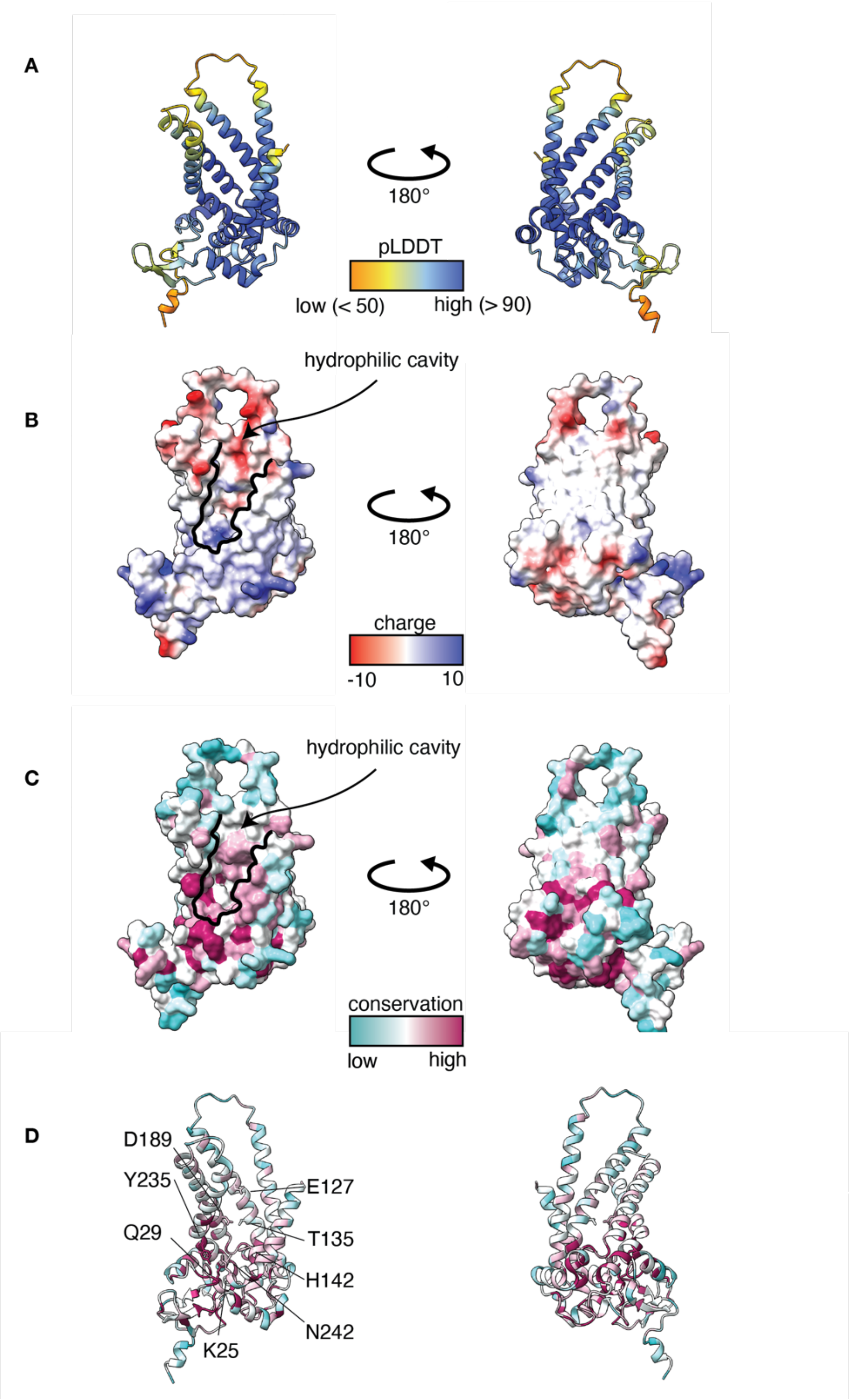

Fig. S19. The AlphaFold2 model of human MTCH2 predicts the presence of a conserved hydrophilic groove within the membrane. (A) Two views of a cartoon rendering of the Alphafold2 predicted MTCH2 model from the plane of the membrane, colored by model confidence (pLDDT). **(B)** As in (A) with surface representation of the predicted model, colored by coulombic electrostatic potential calculated by the ChimeraX structural visualization software package (*71*). **(C)** The same model views shown in surface representation and colored by

sequence conservation calculated from the same sequence alignment shown in fig. S16 in ChimeraX. **(D)** The same model views shown in cartoon representation, also colored by sequence conservation as above, and with residues used in mutational analysis shown in stick representation and labeled. Using these models enabled mutational analysis for a potential mechanism of MTCH2 mediated membrane protein insertion involving the conserved hydrophilic groove within the membrane. We reasoned that negatively charged residues lining the middle-to-upper portion of the hydrophilic cavity may be important for the docking of mitochondrial TAs which are enriched for flanking positive charges at their C-termini.

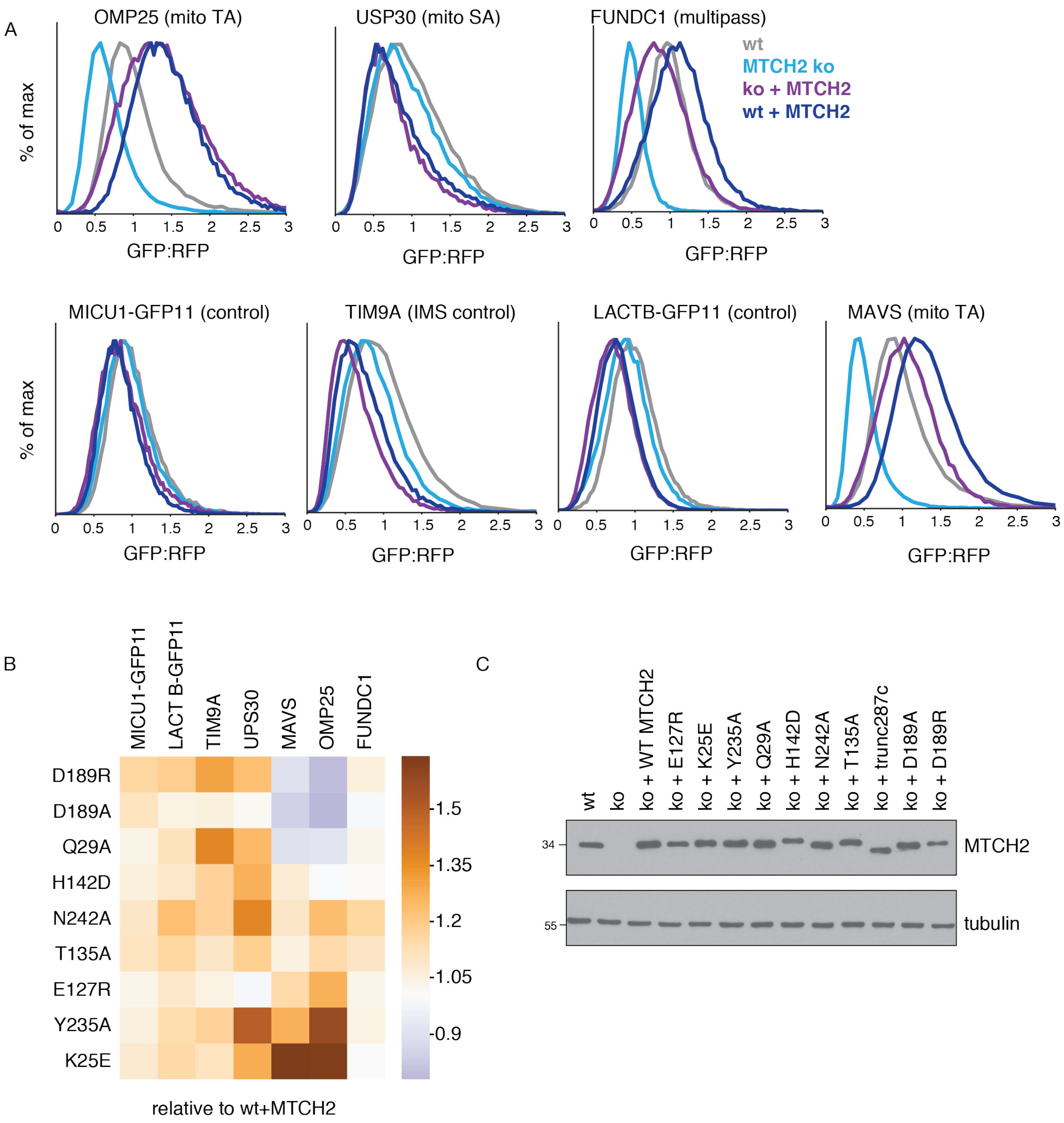

Fig. S20. Point mutants to conserved polar and charged residues in MTCH2's TMDs affect integration of mitochondrial TAs. Using our fluorescent reporter assay (Fig. 1B), we tested the effects of expression of a series of MTCH2 mutants on the biogenesis of the indicated mitochondrial proteins. In **(A)** we depict the effects of ectopic expression of wild-type MTCH2 in either wild-type (wt) or MTCH2 knockout (ko). Because MTCH2 in this system is overexpressed compared to endogenous levels, it is sufficient to drive increased integration of MTCH2 dependent, but not independent, substrates into the mitochondrial outer membrane. **(B)** A heat map, summarizing the effects of expression of the indicated MTCH2 mutants on top of wild-type cells, which are expressing a functional endogenous copy of MTCH2. Depicted is the fold-change in insertion for the indicated substrates upon ectopic expression of each MTCH2

mutant compared to wild-type MTCH2. Mutants were selected based on their conservation and localization within the hydrophilic groove of MTCH2 (Fig. 4C, fig. S19). A matched experiment performed in MTCH2 knockout cells is summarized in Fig. 4C. **(C)** Immunoblots showing the expression of wild-type MTCH2 and the indicated point mutants in a K562 MTCH2 ko background relative to wild-type cells. We note the differential effect of some mutants on TA substrates (MAVS, OMP25) compared to a multipass substrate (FUNDC1), which may indicate that FUNDC1 expression is itself regulated, and thus does not accumulate over endogenous levels. Alternatively, it may be that TAs and multipass proteins utilize different regions of MTCH2 for insertion, as was observed for the EMC, where certain mutations have differential effects on TA and multipass substrates (*24*, *25*).

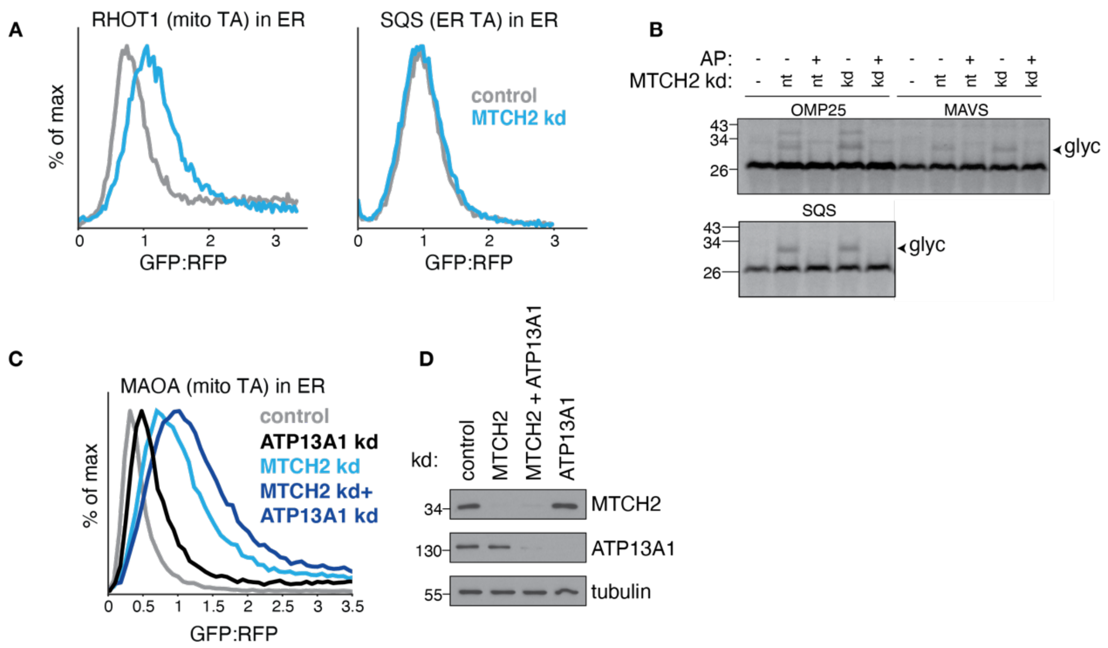

Fig. S21. Depletion of MTCH2 causes increased mislocalization of mitochondrial TAs to the ER. **(A)** Cell lines expressing GFP1-10 in the ER lumen were used to monitor mislocalization to the ER of mitochondrial TAs fused to a C-terminal GFP11 using a similar strategy to that previously described (*72*). Flow cytometry analysis of insertion into the ER of a mitochondrial TA (RHOT1) or an ER resident protein (SQS) in cells depleted of MTCH2. **(B)** Mislocalization of mitochondrial TAs in wild-type versus MTCH2 depleted cells was analyzed in vitro by appending a C-terminal opsin tag to their C-termini. The substrates were translation in reticulocyte lysate, puromycin treated and then mixed with semi-permeabilized cells. ER localization was detected as a glycosylated species ('glyc'). Acceptor peptide (AP) was used to prevent glycosylation, thereby confirming that the higher molecular weight band corresponded to a glycosylated species. Non-TA and ER resident TA controls were used to confirm the effect was specific to mislocalized mitochondrial TAs. **(C)** As in (A) but MTCH2 was depleted alongside the ER quality control factor ATP13A1. **(D)** Blots depicting MTCH2 and ATP13A1 levels in cells used for reporter assays in (C).

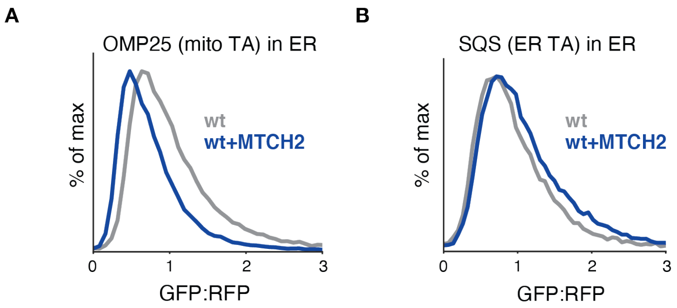

Fig. S22. MTCH2 overexpression results in less mislocalization of mitochondrial TAs to the ER. As in fig. S21 but under conditions where MTCH2 is over-expressed. Note that there is a baseline of mislocalized OMP25-GFP11 reporter in the ER, likely due to its being a dual localized TA protein and over-expression of the construct.

Supplementary Tables

Table S1. Genome-wide CRISPRi screen for mitochondrial TA integration

Table S2. Mass-spectrometry analysis of steady state levels of mitochondrial proteins K562 MTCH2 knockdown cells compared to wild-type cells

Table S3. Mass-spectrometry analysis of steady state levels of mitochondrial proteins K562 MTCH2 knockout cells compared to wild-type cells

Table S4. Mass spectrometry analysis of factors physically associated with the mitochondrial TA protein OMP25

References and Notes

1. Friedman, J. R., Nunnari, J., Mitochondrial form and function. *Nature* **505**, 335 (2014).
2. Wang, W., Zhao, F., Ma, X., Perry, G., Zhu, X., Mitochondria dysfunction in the pathogenesis of Alzheimer's disease: recent advances. *Mol Neurodegener* **15**, 30 (2020).
3. Bose, A., Beal, M. F., Mitochondrial dysfunction in Parkinson's disease. *J Neurochem* **139 Suppl 1**, 216 (2016).
4. Vyas, S., Zaganjor, E., Haigis, M. C., Mitochondria and Cancer. *Cell* **166**, 555 (2016).
5. N, W., N, P., Mitochondrial Machineries for Protein Import and Assembly *The Annual Review of Biochemistry* (2017).
6. Doan, K. N. *et al.*, The Mitochondrial Import Complex MIM Functions as Main Translocase for α-Helical Outer Membrane Proteins. *Cell Rep* **31**, 107567 (2020).
7. Vitali, D. G. *et al.*, Independent evolution of functionally exchangeable mitochondrial outer membrane import complexes *eLife* **7**, (2018).
8. Setoguchi, K., Otera, H., Mihara, K., Cytosolic factor- and TOM-independent import of C-tail-anchored mitochondrial outer membrane proteins. *EMBO J* **25**, 5635 (2006).
9. Gilbert, L. A. *et al.*, Genome-Scale CRISPR-Mediated Control of Gene Repression and Activation. *Cell* **159**, 647 (2014).
10. Le Vasseur, M. *et al.*, Genome-wide CRISPRi screening identifies OCIAD1 as a prohibitin client and regulatory determinant of mitochondrial Complex III assembly in human cells. *Elife* **10**, e67624 (2021).
11. Coukos, R. *et al.*, An engineered transcriptional reporter of protein localization identifies regulators of mitochondrial and ER membrane protein trafficking in high-throughput CRISPRi screens. *Elife* **10**, e69142 (2021).

12. Itakura, E. *et al.*, Ubiquilins Chaperone and Triage Mitochondrial Membrane Proteins for Degradation. *Mol Cell* **63**, 21 (2016).
13. Ruprecht, J. J., Kunji, E. R. S., The SLC25 Mitochondrial Carrier Family: Structure and Mechanism. *Trends Biochem Sci* **45**, 244 (2020).
14. Labbé, K. *et al.*, The modified mitochondrial outer membrane carrier MTCH2 links mitochondrial fusion to lipogenesis. *J Cell Biol* **220**, e202103122 (2021).
15. Rottiers, V. *et al.*, MTCH2 is a conserved regulator of lipid homeostasis. *Obesity (Silver Spring)* **25**, 616 (2017).
16. Zaltsman, Y. *et al.*, MTCH2/MIMP is a major facilitator of tBID recruitment to mitochondria. *Nat Cell Biol* **12**, 553 (2010).
17. Replogle, J. M. *et al.*, Mapping information-rich genotype-phenotype landscapes with genome-scale Perturb-seq. *Cell* **185**, 2559 (2022).
18. Fry, M. Y., Saladi, S. M., Cunha, A., Clemons, W. M., Sequence-based features that are determinant for tail-anchored membrane protein sorting in eukaryotes. *Traffic* **22**, 306 (2021).
19. McKenna, M. J. *et al.*, The endoplasmic reticulum P5A-ATPase is a transmembrane helix dislocase. *Science* **369**, eabc5809 (2020).
20. Palmieri, F., The mitochondrial transporter family SLC25: identification, properties and physiopathology. *Mol Aspects Med* **34**, 465 (2013).
21. Shi, X. *et al.*, Combinatorial GxGxE CRISPR screen identifies SLC25A39 in mitochondrial glutathione transport linking iron homeostasis to OXPHOS. *Nat Commun* **13**, 2483 (2022).

22. Jumper, J. *et al.*, Highly accurate protein structure prediction with AlphaFold. *Nature* **596**, 583 (2021).

23. Benito, A., Grillot, D., Nuñez, G., Fernández-Luna, J. L., Regulation and Function of Bcl-2 During Differentiation-Induced Cell Death in HL-60 Promyelocytic Cells *American Journal of Pathology* **146**, 481 (1995).

24. Pleiner, T. *et al.*, Structural basis for membrane insertion by the human ER membrane protein complex. *Science* **369**, 433 (2020).

25. Miller-Vedam, L. E. *et al.*, Structural and mechanistic basis of the EMC-dependent biogenesis of distinct transmembrane clients. *Elife* **9**, e62611 (2020).

26. Bai, L., You, Q., Feng, X., Kovach, A., Li, H., Structure of the ER membrane complex, a transmembrane-domain insertase. *Nature* **584**, 475 (2020).

27. Wu, X. *et al.*, Structural basis of ER-associated protein degradation mediated by the Hrd1 ubiquitin ligase complex. *Science* **368**, eaaz2449 (2020).

28. Kumazaki, K. *et al.*, Structural basis of Sec-independent membrane protein insertion by YidC. *Nature* **509**, 516 (2014).

29. Rao, M. *et al.*, Multiple selection filters ensure accurate tail-anchored membrane protein targeting *Elife* (2016).

30. Karch, C. M., Ezerskiy, L. A., Bertelsen, S., Alzheimer's Disease Genetics Consortium, A. D. G. C., Goate, A. M., Alzheimer's Disease Risk Polymorphisms Regulate Gene Expression in the ZCWPW1 and the CELF1 Loci. *PLoS One* **11**, e0148717 (2016).

31. Allen, M. *et al.*, Late-onset Alzheimer disease risk variants mark brain regulatory loci. *Neurol Genet* **1**, e15 (2015).

32. Escott-Price, V. *et al.*, Gene-wide analysis detects two new susceptibility genes for Alzheimer's disease. *PLoS One* **9**, e94661 (2014).

33. Riggs, P., Expression and purification of recombinant proteins by fusion to maltose-binding protein *Molecular Biotechnology* (2000).

34. Brambillasca, S. *et al.*, Transmembrane topogenesis of a tail-anchored protein is modulated by membrane lipid composition. *EMBO J* **24**, 2533 (2005).

35. Chen, J. J. *et al.*, Compromised function of the ESCRT pathway promotes endolysosomal escape of tau seeds and propagation of tau aggregation. *J Biol Chem* **294**, 18952 (2019).

36. Jost, M. *et al.*, Combined CRISPRi/a-Based Chemical Genetic Screens Reveal that Rigosertib Is a Microtubule-Destabilizing Agent. *Mol Cell* **68**, 210 (2017).

37. Chitwood, P. J., Juszkiewicz, S., Guna, A., Shao, S., Hegde, R. S., EMC Is Required to Initiate Accurate Membrane Protein Topogenesis. *Cell* **175**, 1507 (2018).

38. Guna, A., Volkmar, N., Christianson, J. C., Hegde, R. S., The ER membrane protein complex is a transmembrane domain insertase. *Science* **359**, 470 (2018).

39. Cabantous, S., Terwilliger, T. C., Waldo, G. S., Protein tagging and detection with engineered self-assembling fragments of green fluorescent protein. *Nat Biotechnol* **23**, 102 (2005).

40. Kamiyama, D. *et al.*, Versatile protein tagging in cells with split fluorescent protein. *Nat Commun* **7**, 11046 (2016).

41. Hung, V. *et al.*, Proteomic mapping of the human mitochondrial intermembrane space in live cells via ratiometric APEX tagging. *Mol Cell* **55**, 332 (2014).

42. Replogle, J. M. *et al.*, Combinatorial single-cell CRISPR screens by direct guide RNA capture and targeted sequencing. *Nat Biotechnol* **38**, 954 (2020).

43. Kanarek, N. *et al.*, Histidine catabolism is a major determinant of methotrexate sensitivity. *Nature* **559**, 632 (2018).

44. Vera Rodriguez, A., Frey, S., Görlich, D., Engineered SUMO/protease system identifies Pdr6 as a bidirectional nuclear transport receptor. *J Cell Biol* **218**, 2006 (2019).

45. Frey, S., Görlich, D., A new set of highly efficient, tag-cleaving proteases for purifying recombinant proteins. *J Chromatogr A* **1337**, 95 (2014).

46. Pleiner, T. *et al.*, Nanobodies: site-specific labeling for super-resolution imaging, rapid epitope- mapping and native protein complex isolation *Elife* (2015).

47. Chin, J. W. *et al.*, Addition of p-azido-L-phenylalanine to the genetic code of Escherichia coli. *J Am Chem Soc* **124**, 9026 (2002).

48. Götzke, H. *et al.*, The ALFA-tag is a highly versatile tool for nanobody-based bioscience applications *Nature Communications* (2019).

49. Canaj, H. *et al.*, Deep profiling reveals substantial heterogeneity of integration outcomes in CRISPR knock-in experiments *Biorxiv* (2019).

50. Horlbeck, M. A., Gilbert, L. A., Villalta, J. E., Kampmann, M., Weissman, J. S., Compact and highly active next- generation libraries for CRISPR-mediated gene repression and activation *Elife* (2016).

51. Pleiner, T., Bates, M., Görlich, D., A toolbox of anti-mouse and anti-rabbit IgG secondary nanobodies. *J Cell Biol* **217**, 1143 (2018).

52. Richter-Dennerlein, R. *et al.*, DNAJC19, a mitochondrial cochaperone associated with cardiomyopathy, forms a complex with prohibitins to regulate cardiolipin remodeling. *Cell Metab* **20**, 158 (2014).

53. Sharma, A., Mariappan, M., Appathurai, S., Hegde, R. S., In vitro dissection of protein translocation into the mammalian endoplasmic reticulum *Methods Mol Biol* (2010).

54. Morgenstern, M. *et al.*, Quantitative high-confidence human mitochondrial proteome and its dynamics in cellular context. *Cell Metab* **33**, 2464 (2021).

55. Breiman, L., Random Forests *Machine Learning* (2001).

56. Rath, S. *et al.*, MitoCarta3.0: an updated mitochondrial proteome now with sub-organelle localization and pathway annotations. *Nucleic Acids Res* **49**, D1541 (2021).

57. Go, C. D. *et al.*, A proximity-dependent biotinylation map of a human cell. *Nature* **595**, 120 (2021).

58. Wagner, F. *et al.*, Armadillo repeat-containing protein 1 is a dual localization protein associated with mitochondrial intermembrane space bridging complex. *PLoS One* **14**, e0218303 (2019).

59. Gok, M. O., Friedman, J. R., The outer mitochondrial membrane protein TMEM11 is a novel negative regulator of BNIP3/BNIP3L-dependent receptor-mediated mitophagy *Biorxiv* (2022).

60. Mariappan, M. *et al.*, The mechanism of membrane-associated steps in tail-anchored protein insertion. *Nature* **477**, 61 (2011).

61. Eddy, S. R., Accelerated Profile HMM Searches. *PLoS Comput Biol* **7**, e1002195 (2011).

62. Mistry, J. *et al.*, Pfam: The protein families database in 2021. *Nucleic Acids Res* **49**, D412 (2021).

63. Edgar, R. C., MUSCLE: multiple sequence alignment with high accuracy and high throughput. *Nucleic Acids Res* **32**, 1792 (2004).

64. Robert, X., Gouet, P., Deciphering key features in protein structures with the new ENDscript server. *Nucleic Acids Res* **42**, W320 (2014).

65. Dimmer, K. S. *et al.*, A crucial role for Mim2 in the biogenesis of mitochondrial outer membrane proteins. *J Cell Sci* **125**, 3464 (2012).

66. Becker, T. *et al.*, Biogenesis of the mitochondrial TOM complex: Mim1 promotes insertion and assembly of signal-anchored receptors. *J Biol Chem* **283**, 120 (2008).

67. Waizenegger, T., Schmitt, S., Zivkovic, J., Neupert, W., Rapaport, D., Mim1, a protein required for the assembly of the TOM complex of mitochondria. *EMBO Rep* **6**, 57 (2005).

68. Thornton, N. *et al.*, Two modular forms of the mitochondrial sorting and assembly machinery are involved in biogenesis of alpha-helical outer membrane proteins. *J Mol Biol* **396**, 540 (2010).

69. Hulett, J. M. *et al.*, The transmembrane segment of Tom20 is recognized by Mim1 for docking to the mitochondrial TOM complex. *J Mol Biol* **376**, 694 (2008).

70. Käser, S. *et al.*, Outer membrane protein functions as integrator of protein import and DNA inheritance in mitochondria. *Proc Natl Acad Sci U S A* **113**, E4467 (2016).

71. Pettersen, E. F. *et al.*, UCSF ChimeraX: Structure visualization for researchers, educators, and developers. *Protein Sci* **30**, 70 (2021).

72. Inglis, A. J., Page, K. R., Guna, A., Voorhees, R. M., Differential Modes of Orphan Subunit Recognition for the WRB/CAML Complex. *Cell Rep* **30**, 3691 (2020).
